# TRIM44, a Novel Prognostic Marker, Supports the Survival of Proteasome-Resistant Multiple Myeloma Cells

**DOI:** 10.3390/cells13171431

**Published:** 2024-08-26

**Authors:** Trung Vu, Yuqin Wang, Annaliese Fowler, Anton Simieou, Nami McCarty

**Affiliations:** 1 Brown Foundation Institute of Molecular Medicine for the Prevention of Human Diseases (IMM), The University of Texas-Health Science Center at Houston, Houston, TX 77021, USA; trung.vu.1@uth.tmc.edu (T.V.); yuqin.wang@uth.tmc.edu (Y.W.); 2 The Department of Biomedical Engineering, Texas A&M University, Houston, TX 77030, USA; afowl@tamu.edu; 3 The Department of Biomedical Engineering, The University of Texas at Austin, Austin, TX 78712, USA; asimieou@texas.edu

**Keywords:** multiple myeloma, unfolded protein response, autophagy

## Abstract

TRIM44, a tripartite motif (TRIM) family member, is pivotal in linking the ubiquitin-proteasome system (UPS) to autophagy in multiple myeloma (MM). However, its prognostic impact and therapeutic potential remain underexplored. Here, we report that TRIM44 overexpression is associated with poor prognosis in a Multiple Myeloma Research Foundation (MMRF) cohort of 858 patients, persisting across primary and recurrent MM cases. TRIM44 expression notably increases in advanced MM stages, indicating its potential role in disease progression. Single-cell RNA sequencing across MM stages showed significant TRIM44 upregulation in smoldering MM (SMM) and MM compared to normal bone marrow, especially in patients with t(4;14) cytogenetic abnormalities. This analysis further identified high TRIM44 expression as predictive of lower responsiveness to proteasome inhibitor (PI) treatments, underscoring its critical function in the unfolded protein response (UPR) in TRIM44-high MM cells. Our findings also demonstrate that TRIM44 facilitates SQSTM1 oligomerization under oxidative stress, essential for its phosphorylation and subsequent autophagic degradation. This process supports the survival of PI-resistant MM cells by activating the NRF2 pathway, which is crucial for oxidative stress response and, potentially, other chemotherapy-induced stressors. Additionally, TRIM44 counters the TRIM21-mediated suppression of the antioxidant response, enhancing MM cell survival under oxidative stress. Collectively, our discoveries highlight TRIM44’s significant role in MM progression and resistance to therapy, suggesting its potential value as a therapeutic target.

## 1. Introduction

Multiple myeloma (MM), an incurable cancer originating from plasma B cells, is defined by its antibody secretion and accumulation within the bone marrow [1]. This accumulation leads to severe consequences, including bone destruction, anemia, hypercalcemia, renal insufficiency, and interference with normal blood cell production [2]. MM predominantly affects older individuals, where its proliferating cells significantly impact skeletal functions [3].

The introduction of proteasome inhibitors (PI), notably Bortezomib—the first FDA-approved drug in this class for treating MM—has marked a significant advancement. These inhibitors disrupt the ubiquitin–proteasome system, halting the breakdown of proteins that regulate cell cycles and resulting in the accumulation of misfolded/unfolded proteins in the endoplasmic reticulum (ER) [4]. Consequently, they trigger apoptosis, effectively inducing cell death, suppressing proliferation, and increasing tumor cell sensitivity to conventional therapies. Bortezomib, specifically, has shown up to 70% anticancer activity in MM cell lines by selectively and reversibly inhibiting the 26S proteasome’s chymotryptic activity [5]. Its action leads to the accumulation and aggregation of misfolded proteins within the endoplasmic reticulum (ER), activating the unfolded protein response (UPR) through key ER-resident proteins: PKR-like ER kinase (PERK), Inositol-Requiring Enzyme 1 (IRE1), and Activating Transcription Factor 6 (ATF6) [6]. This activation triggers a complex cascade, wherein PERK phosphorylates the α subunit of eIF2α, diminishing overall protein synthesis while selectively boosting the translation of specific mRNAs such as Activating Transcription Factor 4 (ATF4) [7]. ATF4 induces a range of genes involved in resolving ER stress, including those encoding amino acid transporters and ER resident chaperones. These targets manage oxidative stress, amino acid synthesis, and vital survival processes, including autophagy facilitated by the eIF2α/PERK pathway [8]. This ER stress-induced autophagy plays a crucial role in MM cell survival, highlighting a significant route through which resistance to Bortezomib may emerge.

Autophagy, a fundamental cellular process for degrading and recycling long-lived proteins and damaged cytoplasmic organelles, is initiated through the formation of autophagosomes [9]. These double-membrane vesicles encapsulate cytoplasmic material for degradation within lysosomes. A vital component of this process is the Microtubule-associated protein 1 light chain 3B (LC3B), which undergoes conversion from LC3-I to LC3-II during autophagosome formation [10]. Elevated protein synthesis in MM cells intensifies ER and oxidative stress, increasing reliance on autophagy for maintaining cellular balance. During selective autophagy, ubiquitinated cytoplasmic aggregates and damaged organelles are targeted for degradation, with adaptor proteins like Sequestosome-1 (SQSTM1/p62) playing a key role in their recognition and processing. SQSTM1 not only plays a critical role in autophagosome assembly but also serves as a central component of the anti-oxidative stress response. This dual function may contribute to the survival of multiple myeloma (MM) cells and add complexity to the mechanisms underlying Bortezomib resistance. SQSTM1 plays an important role in anti-oxidative stress responses through the regulation of the NF-E2-related factor 2 (NFE2)-Kelch-like ECH-associated protein 1 (KEAP1) pathway [11]. The NFE2-KEAP1 pathway operates as a fundamental defense against oxidative stress [12]. NRF2, a transcription factor, controls both the basal and induced expression of an array of antioxidant response element (ARE)-dependent genes, thereby regulating the physiological and pathological outcomes of oxidant exposure. KEAP1, the primary regulator of NRF2, contains five domains essential for inhibiting NRF2 activity [13]. SQSTM1 acts as a cellular stress sensor for autophagy deficiency and DNA damage, sequestering KEAP1 and preventing it from suppressing NRF2 activity. As a result, NRF2 accumulates in the nucleus, where it activates an effective antioxidant response, protecting cancer cells from oxidative damage [14].

TRIM44, a member of the tripartite motif (TRIM) family, is notably overexpressed in quiescent MM cells from the osteoblastic niches of the bone marrow [15]. Our research reveals that TRIM44, a deubiquitination enzyme, activates autophagy by promoting SQSTM1 oligomerization and plays a crucial role in aggregate removal by increasing autophagy flux [16]. Hence, TRIM44 emerges as a potential therapeutic target in MM, linking the ubiquitin–proteasome system (UPS) with autophagy processes. We also reported that TRIM44 enhances the DNA damage response and repair by preventing the degradation of key proteins through the inhibition of SQSTM1 localization to the nucleus via oligomerization [17]. 

In this report, we found that TRIM44 overexpression correlates with poor prognosis and disease progression in MM patients, particularly those with the cytogenetic abnormality of t(4;14) translocation. The pattern of expression is similar to the markers for t(4;14), such as Wolf–Hirschhorn Syndrome Candidate 1 (WHSC1) and the fibroblast growth factor receptor 3 (FGFR3). Analysis of several single-cell RNAseq datasets shows that TRIM44 expression predicts groups less responsive to proteasome inhibitor (PI) treatment. TRIM44-high optimal responder groups show suppressed pathways related to the unfolded protein response, a crucial downstream signal for TRIM44-high MM cells. Further investigation into the molecular mechanisms underlying PI resistance in TRIM44-high MM cells reveals that Bortezomib treatment induces reactive oxygen species (ROS) stress. Our studies further reveal the molecular mechanisms underlying this process; TRIM44 enhances SQSTM1/p62 oligomerization in response to oxidative stress, thereby increasing substrate degradation via autophagy. Moreover, TRIM44-mediated SQSTM1 oligomerization through phosphorylation at S349 boosts KEAP1 sequestration and NRF2 signaling, supporting PI-resistant MM cell survival. Collectively, our research uncovers new mechanisms underlying PI resistance in MM cells and the critical roles of the UPR-TRIM44-autophagy link in this context.

## 2. Materials and Methods

### 2.1. Cell Lines

HeLa, U266, and RPMI cell lines are from ATCC (Rockland, MD, USA). We verified these cell lines via STR profiling (short tandem repeat DNA analysis). Cell lines that contained knockout or overexpression constructs were not passaged beyond ~8 generations (1–1.5 months). HeLa cells were cultured in Dulbecco’s Modified Eagle’s medium (Corning, Steuben County, NY, USA, 10–013-CM) with 10% FBS (Peak serum, PS-FB1). U266 cells were cultured in RPMI medium (GE Healthcare Life Sciences, Chicago, IL, USA, SH30255.01) with 10% FBS. We generated cells in which TRIM44 was overexpressed (TRIM44[OE]) or knocked down (TRIM44[KD]) via a lentivirus. Control cells were infected with relevant control vectors corresponding to the infecting virus vector (TRIM44[OE-CON] or TRIM44[KD-CON]). Lentivirus was packaged using transfer vector (OHS5898-202620525 or RHS4430-200177847), viral packaging (psPAX2), and viral envelope (pMD2G) in 293FT cells.

### 2.2. Antibodies

Anti-TRIM44 polyclonal antibody (Proteintech Group, Solana Beach, CA, USA, 11511-1-AP); anti-Ub antibody (Biolegend, San Diego, CA, USA, 646301); anti-mCherry antibody (ThermoFisher, Plainville, MA, USA, PA5-34974); anti-GFP antibody (Santa Cruz Biotechnology, Santa Cruz, CA, USA, sc-9996); anti-β-Actin antibody (Santa Cruz Biotechnology, Santa Cruz, CA, USA, sc-47778), anti-HA antibody (Santa Cruz Biotechnology, Santa Cruz, CA, USA, sc-805); anti-LC3B antibody (Cell Signaling Technology, Danvers, Massachusetts, USA, 3868); anti-p62 antibody (Cell Signaling Technology, 88588); anti-p62 antibody (Cell Signaling Technology, 88588); anti-GAPDH antibody (Invitrogen, Carlsbad, CA, USA, 39-8600); anti-KEAP1 antibody (Invitrogen, (Invitrogen, Carlsbad, CA, USA, MA5-17106); Alexa Fluor 594 donkey anti-rabbit IgG (H + L) (Invitrogen, (Invitrogen, Carlsbad, CA, USA, A21207); Alexa Fluor 546 goat anti-mouse IgG (H + L) (Invitrogen, (Invitrogen, Carlsbad, CA, USA, A11003).

### 2.3. Reagents

MG132 (Selleck Chemicals, Houston, TX, USA, s2619); 3-MA (Selleck Chemicals, Houston, TX, USA, s2767); pp242 (Selleck Chemicals, Houston, TX, USA, s2218); As[III] (Sigma-Aldrich, Santa Clara, CA, USA, 01969); Cycloheximide (Sigma-Aldrich, Santa Clara, CA, USA, C7698).

### 2.4. Apoptosis Assay

MM cells were treated with 5 µM As[III] and harvested after 24 h. The cells were stained with PE-Annexin V and 7-AAD (BD Bioscience, Milpitas, CA, USA, 559763) and examined with an LSR-II flow cytometer. The percentage of apoptosis was analyzed using FACS Diva software (BD Bioscience, Milpitas, CA, USA).

### 2.5. Confocal Microscopy

The cells were fixed with 4% paraformaldehyde (ThermoFisher, Plainville, MA, USA, AAJ61899AK) and permeabilized with 0.2% triton-X 100 (Sigma-Aldrich, Santa Clara, CA, USA, 93443) and blocked with Animal-Free Blocker (Vector laboratories, Burlingame, CA, USA, SP-5030-250) for 1 h, followed by washing with 1× phosphate buffer saline (PBS) (Corning, Steuben County, NY, USA, 46-013-CM; pH 7.4). The cells were then incubated with indicated antibodies overnight at 4 °C, washed three times with PBS, and incubated with fluorochrome-conjugated secondary antibodies for 1 h at room temperature, washed three times with PBS, and incubated with DRAQ5 (Cell Signaling Technology, 4084) for 30 min. Slides were analyzed by confocal microscopy (Leica TCS SP5, Leica Microsystems, Exton, PA, USA). To quantify indicated protein levels, four or five random images were taken from each slide, and were quantified with Image J 1.52i software (National Institutes of Health).

### 2.6. Immunoprecipitation and Immunoblotting

Cells were lysed in RIPA buffer containing a protease inhibitor (Sigma) and incubated with indicated antibodies (anti-TRIM44 (Proteintech, Solana Beach, CA, USA, 11511-1-AP), anti-HA (Santa Cruz Biotechnology, Santa Cruz, CA, USA, sc-805, and anti-mCherry (ThermoFisher, Plainville, MA, USA, PA5-34974) antibodies) together with the protein A/G plus-agarose immunoprecipitation reagent (Santa Cruz Biotechnology, Santa Cruz, CA, USA, sc-2003) at 4 °C overnight. Immunoprecipitates were eluted with Laemmli sample buffer (Bio-Rad, Hercules, CA, USA) and analyzed using the indicated antibodies.

### 2.7. CHX Chase Assay

MM cells were seeded into 6-well plates at 3 × 10^5^ cells/well density and incubated overnight at 37 °C in a CO_2_ incubator. Cells were treated with 50 μg/mL of cycloheximide (CHX) dissolved in absolute ethanol and harvested in ice-cold phosphate-buffered saline (PBS, pH 7.4) at varying chase points by centrifugation at 2500× *g* for 10 min at 4 °C. Cell pellets were lysed in a lysis buffer. Samples were heated at 95 °C for 10 min.

### 2.8. Cell Lysate Fraction

Cells were lysed in an NP-40-containing lysis buffer (BP-119, Boston Bioproducts, Ashland, MA, USA) supplemented with protease inhibitors mixture (11697498001, Complete; Roche Diagnostics, Mannheim, Germany) and centrifuged at 15,000× *g* into supernatant (Non-aggregates) and pellet (Aggregates) fractions. Both fractions were boiled in a buffer containing 1% SDS and analyzed by Western blot.

### 2.9. RNA Extraction and Real-Time PCR

Total RNA was isolated using the Direct-zolTM RNA MicroPrep kit (Zymo Research, Irvine, CA, USA, R2060), and cDNA was synthesized using random hexamers and a RevertAid RT kit (Thermo Scientific, Plainville, MA, USA, K1691). Gene expression was determined using an ABI 7900 system (Applied Biosystems, Foster City, CA, USA) with SYBR Green MasterMix Plus (Thermo Scientific, K0221) and normalized to ACTB expression. Relative expression was calculated as 2^−(Ct Target−Ct Control)^.

### 2.10. Patient Dataset Analysis

The MMRF RNA-sequencing data were downloaded from the Genomic Data Commons data portal (GDC, https://portal.gdc.cancer.gov, accessed on 2 February 2023). The MMRF clinical data were downloaded from (http://xena.ucsc.edu, accessed on 7 February 2023).

### 2.11. Gene Expression and Mutation Status

Baseline RNAseq data for 16 multiple myeloma cell lines were obtained from the Cancer Cell Line Encyclopedia (CCLE; https://portals.broadinstitute.org/ccle, accessed on 15 February 2023). We extracted the normalized expression levels of TRIM44 for t(4;14)-positive multiple myeloma (MM) cell lines (KMS-11, KMS-34, NCI-H929, LP-1, and OPM-2) and t(4;14)-negative MM cell lines (KMS-12, KMS-26, KMS-27, KMM-1, KMS-20, JJN-3, AMO-1, HuT 102, HuNS1, U266B1, MM1-S, RPMI 8226).

### 2.12. scRNA-Seq Data Analysis

The single-cell RNAseq analyses of GSE193531 were conducted in R 4.3.1. The UMI count matrix of the GSE193531 dataset was downloaded from Gene Expression Omnibus databases (GEO). The human genome (hg38) was used as a reference. Raw gene expression matrices were constructed into a Seurat object and imported into R software by the Seurat R package (version 4.1.0). Cell-level quality control analysis was performed to filter cells by (1) total UMI counts of no more than 1000, (2) gene numbers no higher than 200, or (3) mitochondrial gene percentage of >20%. The expression level of each gene in each cell was normalized using the NormalizeData function and the LogNormalize method. ‘IntegrateData’ was then applied to integrate the datasets using the pre-computed anchors; the integrated dataset was scaled using ‘ScaleData’. PCA and uniform manifold approximation and projection (UMAP) dimension reduction based on the top 20 principal components was performed.

The single-cell RNAseq analyses of the GSE161195 and GSE189460 datasets were conducted in Python 3.11.4. The h5ad files were downloaded from GEO under accession numbers GSE161195 and GSE189460 and were parsed to the portal via a Python script using the Scanpy library. Cells were excluded from the analysis according to the following criteria: (i) low-quality single-cell libraries with <400 detected genes and >10% mitochondrial counts were removed; (ii) cell doublets were identified with the Scrublet Python package. Principal component analysis (PCA) was applied for linear dimensionality reduction with the top 3000 variable genes. Shared nearest neighbor graphs were computed using the scanpy.pp.neighbors function in Scanpy, and cells were clustered using UMAP embedding in two-dimensional space. Differentially expressed genes between groups of cells were identified by a Wilcoxon rank-sum test (padj < 0.05, logFC > 0.1) using get.rank_genes_groups_df function in Scanpy. Plasma cells were identified based on expressing high levels of TNFRSF17, SDC1, SLAMF7, and CD38. Gene expression profiles for SORs and ORs in each cell type were generated using the function scanpy.pp.normlize_total function in Scanpy using scaled normalized counts of the top 3000 variable genes.

### 2.13. Statistical Analysis

The Cox proportional hazards (PH) regression model was used to calculate the hazard ratio (HR) and *p* values. The K-M method was used to create the survival plots, and the log-rank test was used to compare the difference in survival curves. The Wilcoxon rank-sum test was used to test the difference between the distributions of uncensored survival time. A two-tailed *p*-value < 0.05 was considered statistically significant for all tests. In situations of multiple tests, the false discovery rate (FDR) was calculated using the Benjamini and Hochberg method. All analyses were performed using R 4.3.1. Significant differences in mean values were evaluated by Student’s *t*-test (unpaired, two-tailed), with *p* < 0.05 considered significant. Asterisks for *p*-value indicate the following: *—*p* < 0.05, **—*p* < 0.01, ***—*p* < 0.001. The results are expressed as the mean ± SD from at least 3–5 independent experiments.

## 3. Results

### 3.1. High TRIM44 Expression Is Associated with Poor Prognosis in the MMRF Cohort

**Figure 1:**
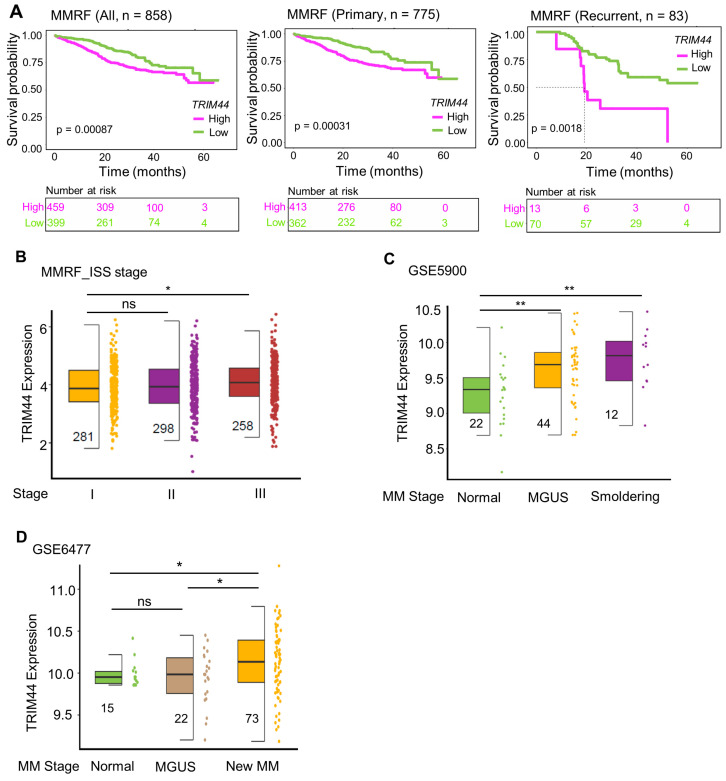
Association of high TRIM44 expression with unfavorable prognosis in MMRF Cohort. (**A**) Relationship between elevated TRIM44 expression and poorer survival outcomes in the MMRF Cohort. Survival analysis based on TRIM44 expression levels (high vs. low) indicates a correlation with overall survival. Each plot displays *p*-values and hazard ratios (HR) derived from a two-sided Log-rank test. The categorization of expression levels is conducted using the “surv_cutpoint” function. The analysis includes primary blood-derived MM (Primary) and recurrent blood-derived MM (Recurrent). (**B**) TRIM44 expression across ISS Stages in MMRF. Boxplots illustrate the variation of TRIM44 mRNA levels across different International Staging System (ISS) stages, with the number of samples for each stage provided. (**C**) TRIM44 expression in the GSE5900 Dataset. This section shows TRIM44 mRNA levels across various disease stages, including data from 22 healthy donors, 44 monoclonal gammopathy of undetermined significance (MGUS) cases, and 12 smoldering myeloma cases. (**D**) TRIM44 levels in the GSE6477 Dataset. TRIM44 mRNA expression is presented across disease stages: 15 healthy donors (Normal), 22 MGUS cases (MGUS), 24 smoldering myelomas (Smoldering), 73 newly diagnosed multiple myeloma cases (New MM), and 28 relapsed multiple myeloma cases (Relapse). *p*-values were determined by a two-sided Wilcoxon rank-sum test or *t*-test and adjusted for multiple comparisons. Significance levels are denoted as * *p* < 0.05, ** *p* < 0.01, ns—not significant.

We initiated our investigation into the prognostic significance of TRIM44 expression levels in MM patients by analyzing data from the Multiple Myeloma Research Foundation (MMRF) cohort, which comprised 858 patients [18]. Our analysis revealed that patients with high levels of TRIM44 expression exhibited significantly worse prognoses compared to those with low TRIM44 expression (*p* < 0.001) (Figure 1A left). Furthermore, the link between high TRIM44 expression and adverse outcomes was consistent across various cell origins. In both primary MM (n = 775) and recurrent MM (n = 83) subgroups, elevated TRIM44 levels were uniformly indicative of a poor prognosis (Figure 1A middle and right).

A multivariate analysis of 10,000 patients identified serum albumin and β-2 microglobulin as reliable prognostic markers, establishing the foundation of the International Staging System (ISS) [19]. Our data show that TRIM44 expression significantly increases in stage III compared to stage I (Figure 1B). MM is a plasma cell malignancy located in the bone marrow [20]. It typically evolves from precursor states such as monoclonal gammopathy of undetermined significance (MGUS) and smoldering multiple myeloma (SMM) [21,22]. Patients with SMM have an annual progression rate of 10%, in contrast to 1% in MGUS patients [23,24]. Currently, prognostic evaluations for MGUS primarily depend on a limited set of clinical markers, such as M-spike, light chains, and percentage of tumor burden [25,26]. To further investigate the potential relationship between TRIM44 and MM progression, we analyzed two independent GEO datasets. The analysis of the GSE5900 cohort revealed a significant increase in TRIM44 expression in both MGUS and SMM precursor states (Figure 1C). Another cohort (GSE6477) demonstrated marked upregulation of TRIM44 in both newly diagnosed MM patients and those with relapsed MM (Figure 1D). Collectively, our data support that TRIM44 not only holds prognostic value in MM but may also play a role in disease progression.

### 3.2. Profile of TRIM44 Expression at Single-Cell Resolution during MM Progression

**Figure 2:**
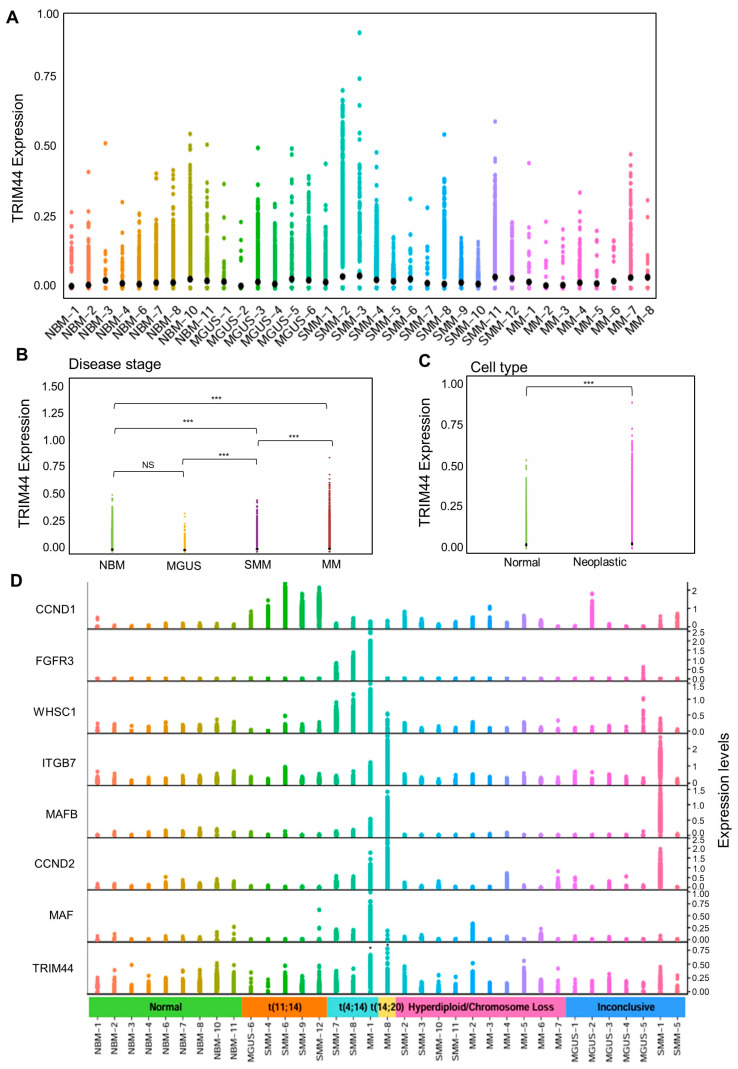
Profiling of TRIM44 expression at single-cell resolution. (**A**) Sample composition overview. A scatter plot displays the distribution of TRIM44 mRNA expression across different samples, including healthy donors (NBM = 9), monoclonal gammopathy of undetermined significance (MGUS, n = 6), smoldering multiple myeloma (SMM, n = 12), and newly diagnosed multiple myeloma (MM, n = 8). Data source: GSE193531. (**B**) TRIM44 expression across disease stages. A scatter plot shows the variation in TRIM44 expression across various disease stages, each represented with a unique color. The plot includes annotations for mean expression values, the number of cells analyzed, and *p*-values obtained from a two-sided Wilcoxon rank-sum test, adjusted for multiple comparisons. Significance indicated by *** *p* < 0.001, ns—not significant. (**C**) TRIM44 expression by cell type. This scatter plot indicates the levels of TRIM44 expression in both normal and malignant cells, with mean expressions highlighted by black dots. Statistical significance was assessed using a Wilcoxon rank-sum test. Significance indicated by *** *p* < 0.001. (**D**) TRIM44 in genetically abnormal samples. Scatter plots illustrate TRIM44 expression in 35 samples identified with chromosomal translocations. The samples are grouped and distinguished by color according to their specific cytogenetic abnormalities. Significance indicated by *** *p* < 0.05.

To delineate the expression profile of TRIM44 in MM patients across different stages of the disease, we analyzed single-cell RNA-sequencing data from 29,387 plasma cells representing 26 samples from patients with MGUS, SMM, or MM in addition to nine samples from normal bone marrow donors (NBM) [27]. The initial analysis aimed to evaluate the distribution of TRIM44 expression across the various samples (Figure 2A). The findings indicated notable variability in TRIM44 expression during the precursor stages of MM (MGUS and SMM) and within MM itself. Significantly, TRIM44 expression was markedly higher in the SMM and MM stages than in NBM (Figure 2B). The relatively lower expression of TRIM44 in MGUS, compared to NBM, may be attributed to the limited cell numbers in these samples. Further examination revealed a significant increase in TRIM44 expression in neoplastic cells over normal cells (Figure 2C).

Translocations involving the immunoglobulin heavy chain region at chromosome 14q32 are observed in approximately 40% of patients with MM [28]. The translocation of oncogenes into this region can lead to their overexpression, contributing to disease initiation, progression, and therapeutic resistance. Known translocations at 14q32 with nonrandom partners include the more commonly observed t(4;14) and t(11;14) translocations (30% of MM patients) and the less common t(14;16), t(6;14), t(8;14), and t(14;20) translocations (≤5% of patients). These translocations are associated with the upregulation of various genes, including D-type cyclins (CCND1, CCND2, and CCND3), MAF family members (MAFB, MAFB, and MAF), WHSC1, and FGFR3, and have been shown to affect patient survival [29]. Given these cytogenetic abnormalities’ known impacts on clinical presentation, progression from precursor stages (MGUS, SMM) to MM, prognosis, and management strategies [26], we further examined the relationship between TRIM44 expression and these translocations. Our findings indicate that the expression pattern of TRIM44 parallels that of markers for the t(4;14) (FGFR3, WHSC1) and t(14;20) (ITGB7, MAFB, and CCND2) (Figure 2D). Patients with high FGFR3 expression typically exhibit poor prognosis and develop resistance to conventional therapies [29]. Similarly, high levels of MAFB protein in MM cells with the t(14;20) translocation are linked to resistance to proteasome inhibitors, with MAFB protein conferring resistance to proteasome inhibitor-induced apoptosis and activation of the caspase family [30]. This indicates that TRIM44 could serve as a valuable prognostic marker for aggressive MM with translocations, implicating TRIM44 expression in the pathogenesis and progression of MM and potentially influencing treatment outcomes. To confirm the overexpression of TRIM44 in t(4;14)-positive multiple myeloma (MM) cells, we analyzed RNA sequencing data from the CCLE. We compared TRIM44 expression levels between t(4;14)-positive and t(4;14)-negative MM cell lines. The analysis revealed that TRIM44 expression is significantly higher in t(4;14)-positive MM cell lines (Figure S1). These results underscore TRIM44’s significant role in the development and advancement of MM.

### 3.3. TRIM44 Levels Strongly Correlate with an Increased Unfolded Protein Response in Patients Exhibiting Low Response to Bortezomib

Despite Bortezomib’s status as a highly effective chemotherapeutic agent for multiple myeloma (MM), resistance to the drug frequently develops, often associated with disease relapse. This resistance is noted even among newly diagnosed MM patients undergoing their initial treatment. Various mechanisms are proposed to underlie cancer cell multidrug resistance, including increased drug excretion, decreased drug uptake, activation of detoxification systems, inhibition of apoptosis, alterations in cell cycle regulation, and changes in drug targets, with the latter gaining significant attention for its role in MM cell resistance to Bortezomib [31]. MM cells, being the most prolific protein-secreting cells, face constant ER stress, predisposing them to readily induce the UPR under Bortezomib treatment [32]. Identifying novel targets involved in these resistance mechanisms is crucial.

**Figure 3:**
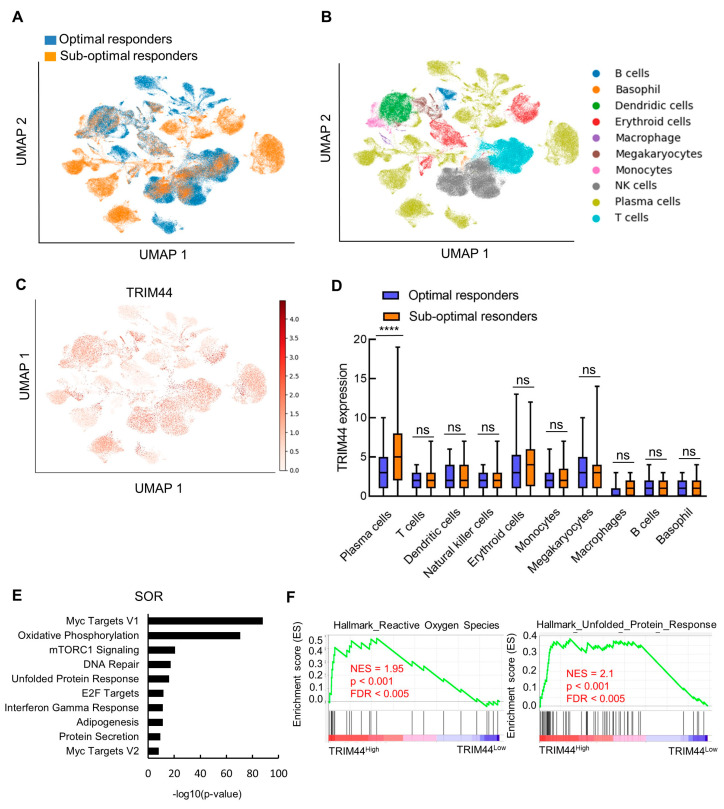
TRIM44 upregulation in Bortezomib-treated MM cells enhances the anti-MM activity of Bortezomib via UPR upregulation. Single-cell RNA sequencing of bone marrow mononuclear cells (MNCs) was analyzed using data from the GSE132465 dataset. These MNCs were isolated from 18 treatment-naive MM patients who subsequently received Bortezomib-based therapies. Patients were classified into two groups based on their response to treatment: optimal responders (ORs) and suboptimal responders (SORs). (**A**) UMAP projection showing major clusters of cells from ORs and SORs. (**B**) Respective cell-type assignments with the same embedding in A. (**C**) UMAP projection showing RNA expression (log-normalized) of TRIM44. (**D**) Boxplots showing normalized expression of TRIM44 in ORs and SORs in different clusters of cells. Significance indicated by **** *p* < 0.0001, ns—not significant. (**E**) Bar plots illustrate the analysis of hallmark gene sets (from the Molecular Signatures Database, mSigDB) based on upregulated differentially expressed genes (DEGs) in TRIM44-high plasma cells from SORs. (**F**) GSEA analysis comparing gene expression profiles from TRIM44-high plasma cells and TRIM44-low plasma cells with signatures of unfolded protein response and reactive oxygen species. FDR, false discovery rate; NES, normalized enrichment score.

To investigate the impact of TRIM44 on patients treated with Bortezomib, we analyzed single-cell RNA-sequencing data from bone marrow-mononuclear cells (BM-MNCs) of 18 treatment-naive MM patients. These patients, who later received Bortezomib-based treatments, were divided into two groups based on their treatment response: ten optimal responders (ORs) and eight suboptimal responders (SORs) [33]. We visualized the data with dimensionality reduction using uniform manifold approximation and projection (UMAP) [34] based on cell types (Figure 3A), treatment response (Figure 3B), and TRIM44 expression (Figure 3C). We analyzed the TRIM44 expression levels in different cell types. The results showed that TRIM44 expression levels are significantly higher in plasma cells of SORs compared to plasma cells of ORs (Figure 3D), while the levels of TRIM44 are not changed in other cell types. Then, clusters were categorized into high-TRIM44 expression and low/negative-TRIM44 expression groups. The top 25% of cells expressing TRIM44 were classified as the high group, while the bottom 25% were classified as the low/negative group. Subsequent differential gene expression analysis identified 2512 upregulated mRNAs in TRIM44-high SOR cells (fold-change > 1.5; *p* < 0.05) and 1033 upregulated mRNAs in TRIM44-high OR cells (fold-change > 1.5; *p* < 0.05). Analysis of the hallmark gene set (mSigDB) in TRIM44-high SOR cells revealed significant enrichment in genes related to Myc targets, oxidative phosphorylation, mTORC1 signaling, DNA repair, and the UPR (Figure 3E). On the other hand, pathway analysis of genes upregulated in TRIM44-high OR cells showed enrichment primarily in the NF-κB pathway, Myc targets, and oxidative phosphorylation (Figure S2). Notably, mTORC1 signaling, DNA repair, and UPR pathways were specifically enriched in TRIM44-high SOR cells but not in TRIM44-high OR cells. Altogether, the results indicate a strong link between elevated TRIM44 levels and an increased UPR in patients exhibiting a low response to Bortezomib treatment, emphasizing the importance of TRIM44 expression and UPR activity in treatment responsiveness. Gene Set Enrichment Analysis (GSEA) confirmed the exclusive enrichment of UPR-related genes in TRIM44-high SORs, underlining TRIM44’s crucial role in the UPR (Figure 3F).

Additionally, we examined another dataset from single-cell RNA sequencing of CD138+ plasma cells from 17 early relapsed patients and 15 newly diagnosed MM patients [35]. Relapse patients were MM patients who received a bortezomib-based induction and failed to achieve a timely response. These patients continued to receive the quadruple anti-myeloma regimen, and non-responders were patients who failed to achieve partial response at 6 months from the enrollment. We visualized the single-cell RNAseq data with dimensionality reduction using uniform manifold approximation and projection (UMAP) based on two markers of plasma cells, XBP1 and SDC1 (CD138) (Figure S3A), treatment response (Figure S3B), and TRIM44 expression (Figure S3C). We analyzed the TRIM44 expression levels in plasma cells. The results showed that TRIM44 expression levels are significantly higher in relapse non-responders compared to responders (Figure S3D). Within the group of non-responders, cells were divided into TRIM44-high and TRIM44-low/negative expression clusters. The top 25% of cells expressing TRIM44 were categorized as the high group, while the bottom 25% were classified as the low/negative group. Analysis of the hallmark gene set (mSigDB) in TRIM44-high cells revealed significant enrichment in genes related to Myc targets, oxidative phosphorylation, mTORC1 signaling, DNA repair, and the UPR. Specifically, genes upregulated in TRIM44-high cells from non-responders were highly enriched in a resistance signature (Figure S3E). Furthermore, we also used RNAseq data from MMRF and divided the patients into TRIM44-high and TRIM44-low/negative expression with the above cutoff. Patients with high expression of TRIM44 have high expression levels of COX7B, CCT3, PDCD5, NDUFB9, CBX3, RPM2, PSMD4, PPIA, and ATF6 genes (Figure S4). These genes, which are associated with the proteasome, ER stress, and UPR pathways, are considered as gene signatures for the resistance of MM [35]. Overall, our findings suggest that high TRIM44 expression is associated with the UPR, which correlates with the resistance of MM cells to Bortezomib.

### 3.4. TRIM44 Is Upregulated and Enhances the Survival of Therapy-Resistant MM Cells Treated with Bortezomib

**Figure 4:**
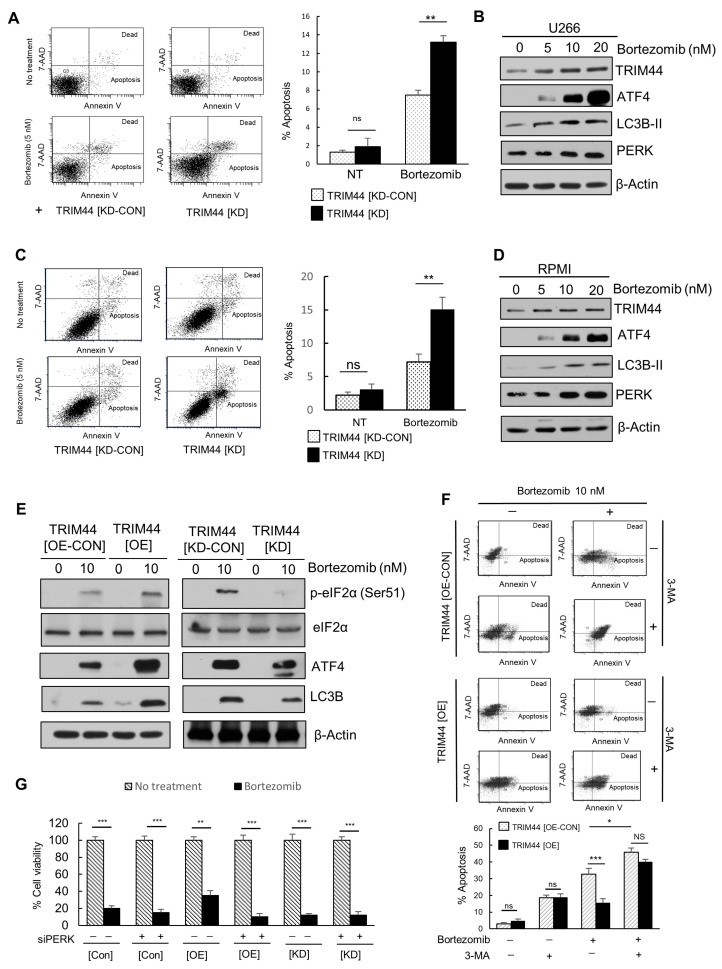
Analysis of TRIM44 expression and apoptotic response in U266 cells treated with Bortezomib. (**A**,**C**) Flow cytometry analysis of apoptosis: U266 cells were treated with 5 nM Bortezomib for 24 h and subsequently stained with PE/7-AAD. The percentage of apoptotic cells was quantified. Significance levels are indicated as ** *p* < 0.01, ns—not significant (unpaired *t*-tests). (**B**,**D**) Western blot analysis of TRIM44 protein levels: U266 (**B**) and RPMI (**D**) cells were exposed to Bortezomib for 24 h. TRIM44 protein levels were then analyzed by Western blot using specific antibodies against TRIM44. (**E**) Western blot analysis of protein phosphorylation: U266 (**C**) or RPMI (**D**) cells overexpressing TRIM44 (TRIM44[OE]) or with TRIM44 knockdown (TRIM44[KD]) were treated with 10 nM Bortezomib for 24 h. Total and phosphorylated proteins were examined by Western blot using indicated antibodies. (**F**) Flow cytometry analysis of apoptosis with autophagy inhibition: U266 cells were treated with 10 nM Bortezomib and 500 μM 3-Methyladenine (3-MA, an autophagy inhibitor) for 24 h, followed by staining with PE/7-AAD. The percentage of apoptotic cells was quantified. Significance levels are denoted as * *p* < 0.05, *** *p* < 0.001 (unpaired *t*-tests). (**G**) Cell proliferation assays following PERK targeting: U266 cells (control [CON], TRIM44-overexpressing [OE], TRIM44 knockdown [KD]) were transfected with siRNA-targeting PERK and treated with 10 nM Bortezomib for 48 h. Significance levels are denoted as ** *p* < 0.01, *** *p* < 0.001.

Based on our analysis of single-cell data (Figure 3 and Figure S3), which suggests TRIM44 roles in Bortezomib resistance, we hypothesized that TRIM44 could be crucial in modulating the drug responses of MM. To explore this hypothesis, we knocked down TRIM44 or overexpressed TRIM44 in MM cell lines such as U266 and PRMI. The changes in TRIM44 protein levels were confirmed by Western blot analysis (Figure S5A). We then treated TRIM44 knockdown U266 and RPMI cells with Bortezomib and analyzed the percentage of apoptotic cells. The results showed a significant increase in apoptosis in the TRIM44 knockdown (TRIM44[KD]) U266 cells in response to Bortezomib treatment (7.5% vs. 13.2%), compared to the control (TRIM44[CON-KD]) (Figure 4A). A similar increase in apoptosis was observed in RPMI cells treated with Bortezomib (Figure 4C). To further confirm the effect of TRIM44 on apoptosis mediated by another proteasome inhibitor, we treated U266 (Figure S5B) and RPMI (Figure S5C) cells with Carfilzomib, a reversible, boronic acid-based proteasome inhibitor. The results showed downregulation in TRIM44 also increased the percentage of apoptotic cells upon the treatment of Carfilzomib. Additionally, to investigate Bortezomib’s effect on TRIM44 expression levels, we treated MM cell lines with varying doses of Bortezomib for 24 h and conducted immunoblot analyses to measure TRIM44 protein levels. Our findings revealed a significant increase in TRIM44 protein levels in a dose-dependent manner in both cell lines (Figure 4B,D and Figure S5C,E).

A previous study by Milani et al. [8] revealed that Bortezomib treatment increases the UPR and autophagy, specifically inducing ATF4 protein levels, which in turn triggers the induction of LC3B. To explore TRIM44’s role in the UPR and autophagy induction in response to Bortezomib treatment, we expressed TRIM44 in U266 cells, treated them with Bortezomib, and analyzed the protein levels of ATF4, phospho-EIF2α, and LC3B. The results indicated that TRIM44 expression elevated the expression levels of ATF4, phosphor-EIF2α, and LC3B in Bortezomib-treated cells, suggesting that TRIM44 overexpression amplifies Bortezomib’s effects on UPR and autophagy (Figure 4C, left panels). We also downregulated TRIM44 in U266 cells by using shRNAs targeting the mRNA of TRIM44 and treated cells with Bortezomib. Our results showed that downregulation in TRIM44 decreased the enhancing effects of Bortezomib treatment on protein levels of ATF4, phosphor-EIF2α, and LC3B, confirming the role of TRIM44 in the Bortezomib-mediated promotion of UPR and autophagy (Figure 4C, right panels). To confirm that TRIM44 enhances the survival of MM cells by inducing autophagy, we treated TRIM44-expressing (TRIM44[OE]) MM cells with Bortezomib, both with and without 3MA (an autophagy inhibitor) and assessed the percentage of apoptotic cells through FACS analysis.

Our findings showed that the percentage of apoptotic cells in TRIM44 overexpressing (TRIM44[OE]) U266 cells was significantly lower in response to Bortezomib treatment (15.7%) compared to the control (TRIM44[CON-OE]) cells (32.5%). However, the addition of 3-Methyladenine (3-MA), an inhibitor that blocks autophagosome formation by inhibiting the class III PI3K complex, mitigated this effect (Figure 4F), highlighting TRIM44’s role in autophagy-mediated survival enhancement in MM cells treated with Bortezomib. To further confirm the role of autophagy in TRIM44-mediated effects, we knocked down ATG5 in TRIM44 overexpressing (TRIM44[OE]) MM cells and control (TRIM44[CON-OE]) MM cells using siRNAs, followed by Bortezomib treatment. We observed that the knockdown of ATG5 abolished the protective effect of TRIM44 overexpression on Bortezomib-induced apoptosis (Figure S5F). To investigate the role of the PERK-EIF2α-ATF4 pathway in TRIM44’s influence on the Bortezomib response, we transfected TRIM44 control, expression ([OE]), and knockdown ([KD]) U266 cells with siRNA targeting PERK. Following this, we treated the cells with Bortezomib and conducted a proliferation assay. The results indicated that downregulating PERK significantly counteracted the Bortezomib resistance observed in [OE] cells, suggesting that simultaneously targeting one of the UPR pathways, specifically PERK, along with TRIM44, synergistically enhances the sensitivity of cells to Bortezomib treatment (Figure 4G).

### 3.5. TRIM44 Promotes the Oligomerization of SQSTM1, Which Is Essential for Its Phosphorylation in Response to Oxidative Stress

**Figure 5:**
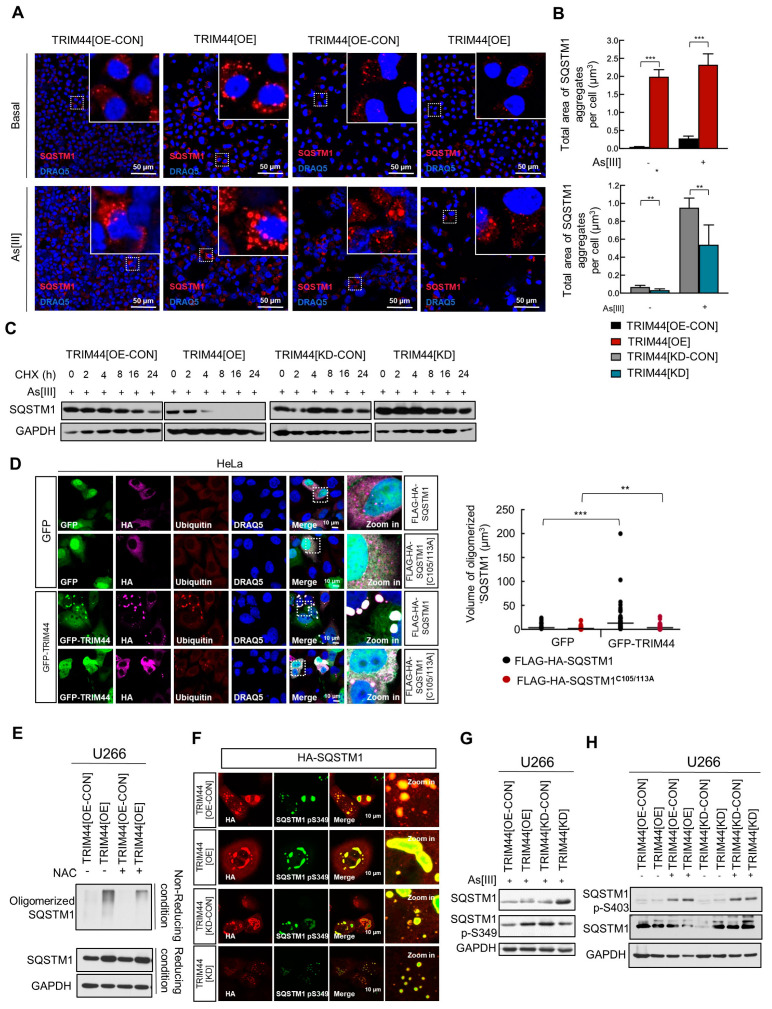
TRIM44 enhances SQSTM1 oligomerization in response to oxidative stress. (**A**) Immunofluorescence staining: U266 cells with TRIM44 overexpression (TRIM44[OE]), TRIM44 overexpression control (TRIM44[OE-CON]), TRIM44 knockdown (TRIM44[KD]), and TRIM44 knockdown control (TRIM44[KD-CON]) were exposed to arsenic trioxide (As[III], 5 μM) for 24 h. Cells were then stained with anti-SQSTM1 antibody (red). Scale bars represent 50 μm. (**B**) Quantification of SQSTM1 aggregates: The total area of SQSTM1 aggregates per cell was quantified across three independent experiments (n = 3), presented as mean ± SD; significance indicated by * *p* < 0.05; ** *p* < 0.01; *** *p* < 0.001. (**C**) Immunoblot analysis: U266 cells with different TRIM44 expressions were treated with cycloheximide (100 μg/mL) and As[III] (5 μM) for specified durations, followed by immunoblotting for SQSTM1. GAPDH served as the loading control. (**D**) Immunofluorescence staining in HeLa cells: Cells were co-transfected with specified plasmids and, after 48 h, stained with anti-HA (magenta), anti-ubiquitin (red), and DRAQ5 (nuclear stain, blue). The volume of oligomerized SQSTM1 was quantified (mean ± SD, n = 3, ** *p* < 0.01, *** *p* < 0.005, unpaired *t*-tests). (**E**) Immunoblot analysis with NAC treatment: U266 cells (TRIM44[OE-CON] and TRIM44[OE]) were treated with N-acetylcysteine (NAC, 5 mM, 24 h) and cross-linked with DSP (0.4 mg/mL) at 4 °C for 2 h. Lysates were prepared under reducing (with β-ME) and non-reducing conditions (without β-ME) and probed for specified antibodies. (**F**) Immunofluorescence staining in HeLa cells: HeLa cells with various TRIM44 expressions were transfected with HA-SQSTM1. After 48 h and exposure to As[III] (5 μM, 24 h), cells were stained with anti-HA (red) and anti-SQSTM1 p-S349 (green). (**G**) Immunoblot analysis for phosphorylation: U266 cells with different TRIM44 expressions were treated with As[III] (5 μM, 24 h) and then immunoblotted for SQSTM1 and SQSTM1 p-S349. (**H**) Immunoblot analysis for additional phosphorylation site: U266 cells with varying TRIM44 expressions treated with or without As[III] (5 μM, 24 h) were immunoblotted for SQSTM1 and SQSTM1 p-S403.

Bortezomib-mediated ER stress leads to the accumulation of reactive oxygen species (ROS), thereby promoting oxidative stress [36]. Several studies have demonstrated that ROS can induce autophagy in response to chemotherapy-induced stress, functioning as a compensatory mechanism to eliminate Bortezomib-induced ROS and resist ER stress-mediated apoptosis [37]. In a recent study, Carroll et al. showed that SQSTM1 detects the saturation of ROS through two oxidation-sensitive cysteine residues, and the oxidation of SQSTM1 facilitates its oligomerization, which in turn activates pro-survival autophagy [14]. Given our findings that TRIM44-mediated autophagy contributes to MM cell resistance to Bortezomib treatment, we further explored the mechanisms by which TRIM44 enhances autophagic activity. With the understanding that Bortezomib induces an oxidative stress response, we treated MM cells with additional oxidative stressors, such as As[III] and H_2_O_2_, and assessed SQSTM1 oligomerization through immunofluorescence staining. We observed a significant increase in SQSTM1 oligomerization in TRIM44-overexpressing (TRIM44[OE]) cells compared to control (TRIM44[OE-CON]) cells under oxidative stress conditions, characterized by larger aggregates (Figure 5A). Knocking down TRIM44 reversed this effect (Figure 5B), underscoring TRIM44’s role in facilitating SQSTM1 oligomerization in response to oxidative stress, a crucial step for its phosphorylation and subsequent autophagic degradation.

To further delineate the effect of TRIM44 on SQSTM1 protein degradation, we minimized the impact of newly synthesized proteins with cycloheximide (CHX), a protein synthesis inhibitor, and monitored the half-life of SQSTM1. SQSTM1, which is continually degraded by autophagy, forms a larger complex with multiple autophagosome cargos before its degradation in the autolysosome. The CHX chase experiments revealed that SQSTM1 degradation was increased in TRIM44[OE] cells compared to TRIM44[OE-CON] cells when treated with As[III], while TRIM44[KD] cells exhibited slower SQSTM1 degradation (Figure 5C). These findings suggest that TRIM44 accelerates the degradation of SQSTM1 under oxidative stress conditions, independent of new protein synthesis. Moreover, inhibiting reactive oxygen species (ROS) with the ROS scavenger N-acetyl-l-cysteine (NAC) reduced SQSTM1 oligomerization levels, indicating that SQSTM1 oligomerization is a reaction to oxidation. Treatment with NAC further decreased SQSTM1 oligomerization levels in TRIM44[OE] cells, underscoring TRIM44’s role in promoting SQSTM1 oligomerization in response to elevated ROS levels (Figure 5D,E).

The two conserved cysteine residues, C105 and C113, located within a disordered region of SQSTM1, are crucial for forming disulphide-linked conjugates (DLC), a process that promotes SQSTM1 oligomerization. SQSTM1’s ability to detect redox changes is vital for its aggregation, particularly in response to oxidative stress [14]. This mechanism serves as a protective response in vertebrates against aging or high oxidative stress conditions [37]. Prior research has demonstrated that SQSTM1 undergoes phosphorylation at S349, S403, or S407 to bind its substrates [38]. Therefore, we delved into the mechanisms connecting redox sensing-induced SQSTM1 oligomerization with phosphorylation, a linkage previously unexplored. Considering that TRIM44 boosts SQSTM1 oligomerization and that such oligomerization is necessary for its phosphorylation, our initial investigation focused on whether TRIM44 could enhance the oligomerization of SQSTM1 phosphorylated at S349. Our findings revealed an increase in phosphorylated SQSTM1 at S349 in TRIM44-overexpressing (TRIM44[OE]) cells but a decrease in TRIM44 knockdown (TRIM44[KD]) cells (Figure 5F). Upon exposure to the oxidative stressor As[III], SQSTM1 phosphorylation at S349 was significantly elevated in TRIM44[OE] cells and diminished in TRIM44[KD] cells (Figure 5G). However, this effect of TRIM44 on SQSTM1 phosphorylation was not evident at the S403 residue (Figure 5H).

### 3.6. TRIM44-Mediated SQSTM1 Oligomerization Sequesters KEAP1 and Activates NRF2, Thereby Enhancing MM Cell Survival under Oxidative Stress

**Figure 6:**
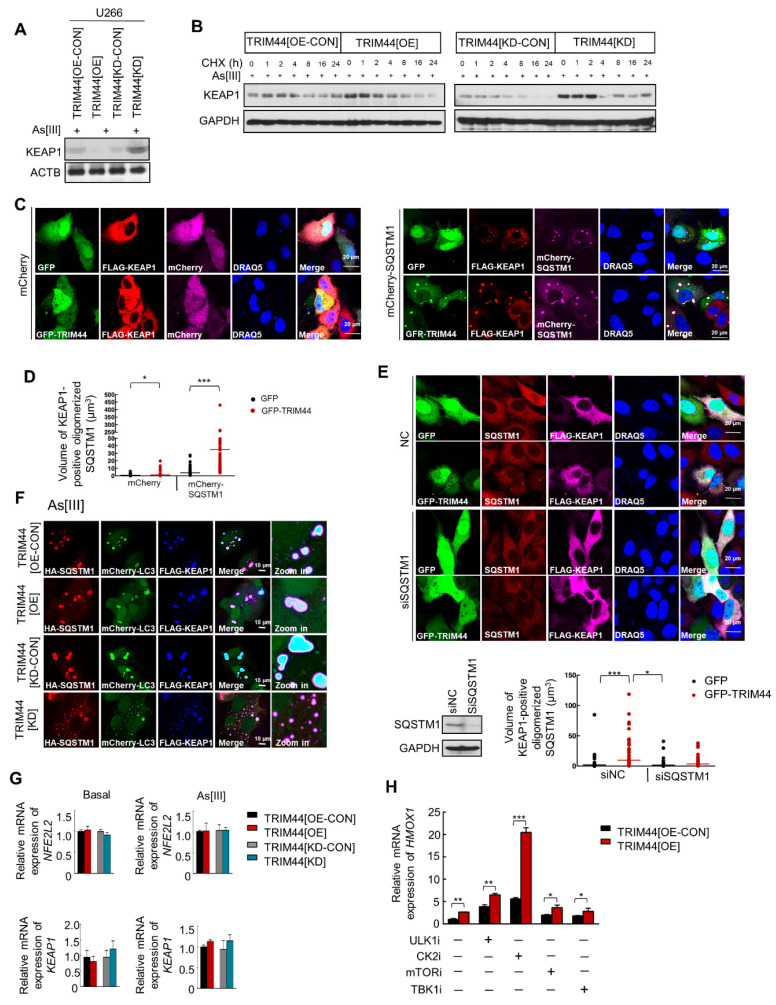
TRIM44 modulates the SQSTM1-KEAP1-NRF2 pathway. (**A**) Immunoblot analysis of KEAP1: Whole-cell lysates from U266 cells with TRIM44 overexpression (TRIM44[OE]), TRIM44 overexpression control (TRIM44[OE-CON]), TRIM44 knockdown (TRIM44[KD]), and TRIM44 knockdown control (TRIM44[KD-CON]) were treated with arsenic trioxide (As[III], 5 μM) for 24 h and analyzed for KEAP1 by immunoblotting. β-actin served as a loading control. (**B**) KEAP1 analysis in HeLa cells: HeLa cells with various TRIM44 expressions were co-treated with As[III] (5 μM) and cycloheximide (CHX, 100 μg/mL) for specified durations. KEAP1 levels were determined by immunoblotting, using GAPDH as a loading control. (**C**,**D**) Immunofluorescence staining for oligomerized SQSTM1: HeLa cells tri-transfected with indicated plasmids were stained with anti-Flag (red) and DRAQ5 (nuclear stain, blue) after 48 h. GFP or GFP-TRIM44 (green) and mCherry or mCherry-SQSTM1 (magenta) indicate protein expression. The volume of oligomerized SQSTM1 was quantified (mean ± SD, n = 3, * *p* < 0.05, *** *p* < 0.001, unpaired *t*-tests). The scale bar represents 20 μm. (**E**) SQSTM1 and KEAP1 interaction in HeLa cells: Following transfection with control (NC) or SQSTM1 siRNA, cells were co-transfected with Flag-Keap1 and either GFP or GFP-TRIM44 for 24 h. Immunostaining was performed for SQSTM1 (red), Flag (magenta), and DRAQ5 (blue). GFP expressions are shown in green. Scale bars represent 20 μm. Immunoblotting for SQSTM1 was conducted with GAPDH as a loading control. Quantification of KEAP1-positive oligomerized SQSTM1 volume was performed (mean ± SD, n = 3, * *p* < 0.05, *** *p* < 0.001, unpaired *t*-tests). (**F**) Immunofluorescence staining with multiple transfections: HeLa cells with different TRIM44 expressions were tri-transfected with HA-SQSTM1, mCherry-LC3, and Flag-Keap1. After 24 h and As[III] treatment (5 μM, 24 h), cells were stained with anti-HA (red) and anti-Flag (blue). mCherry-LC3 expression is depicted in green. Scale bars are 10 μm. (**G**) Nrf2 and Keap1 mRNA levels quantitation: mRNA levels of Nrf2 and Keap1 in U266 cells were measured after As[III] treatment (5 μM, 24 h) via qRT-PCR. Data represent means ± SE from three experiments. (**H**) Quantification of Nrf2 target HMOX1 mRNA levels: U266 cells (TRIM44[OE-CON] and TRIM44[OE]) were treated with inhibitors targeting ULK1 (SBI-0206965, 10 µM), CK2 (CX-4945, 5 µM), mTOR (pp242, 1 µM), and TBK1 (MRT67307, 10 µM) for specified durations before qRT-PCR analysis of HMOX1. Experiments were conducted three times, with data presented as means ± SE (* *p* < 0.05; ** *p* < 0.01; *** *p* < 0.001).

The phosphorylation of oligomerized SQSTM1 at serine residues (S407, S403) within the UBA domain, followed by S349 phosphorylation, increases SQSTM1’s binding affinity for KEAP1. This leads to the release of NRF2 from its interaction with KEAP1, allowing NRF2 to activate various target genes [39]. KEAP1, targeted for autophagic degradation [40], engages with SQSTM1 for this degradation process. Notably, SQSTM1 phosphorylation at serine 349, within the KEAP1-interacting region, strengthens SQSTM1’s interaction with KEAP1 [41]. This suggests that TRIM44 expression may enhance SQSTM1’s capacity to engage with KEAP1, thereby facilitating KEAP1 degradation under oxidative stress conditions. To explore this further, we examined the impact of TRIM44 on KEAP1 protein levels. Our results demonstrated that TRIM44 overexpression led to a decrease in KEAP1 protein levels, while silencing TRIM44 resulted in an increase in KEAP1 (Figure 6A), indicating TRIM44’s involvement in KEAP1 downregulation.

Subsequently, we assessed KEAP1 degradation by treating cells (TRIM44[OE-CON], TRIM44[OE], TRIM44[KD-CON], and TRIM44[KD]) with CHX. After 8 h of CHX treatment, KEAP1 protein levels significantly decreased in TRIM44-overexpressing cells compared to controls, particularly under As[III] treatment, with TRIM44-silencing reversing this effect (Figure 6B). Immunofluorescence analyses revealed that Flag-tagged KEAP1, diffusely distributed in the cytosol of control cells, aggregated into structures associated with SQSTM1 in cells overexpressing TRIM44 (Figure 6C). Moreover, TRIM44 significantly enhanced the sequestration of KEAP1 mediated by SQSTM1 (Figure 6D). Silencing SQSTM1 in TRIM44-overexpressing cells abolished KEAP1 sequestration (Figure 6E), highlighting SQSTM1’s crucial role in TRIM44’s promotion of KEAP1 sequestration. Additionally, TRIM44 facilitated the co-localization of KEAP1 and SQSTM1 with the autophagy-related structure, LC3 (Figure 6F), indicating that TRIM44 promotes KEAP1 sequestration through SQSTM1. Interestingly, no significant change was observed in the relative expression of NRF2 in TRIM44-modified U266 cells with or without As[III] treatment (Figure 6G). However, there was an increase in the relative mRNA expression of the NRF2 target gene HMOX1 with or without the indicated inhibitors (Figure 6H), highlighting the specific regulatory effects of TRIM44 on NRF2 target gene expression under oxidative stress conditions, thereby possibly under chemotherapies.

### 3.7. TRIM44 Counteracts TRIM21-Mediated Suppression of MM Survival under Oxidative Stress

**Figure 7:**
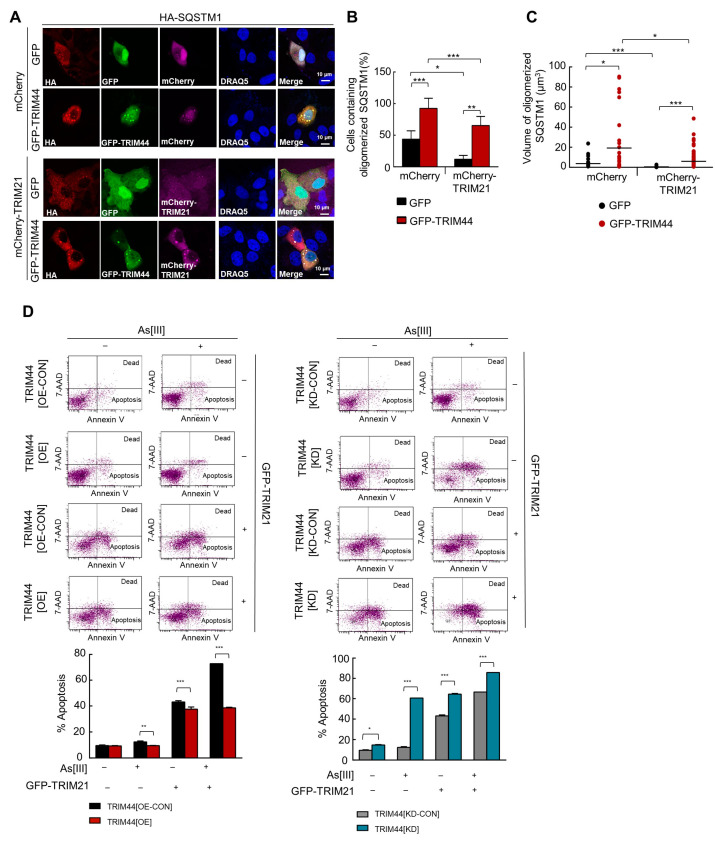
TRIM44 counters TRIM21-mediated cellular sensitivity to oxidative stress. (**A**) Immunofluorescence staining: HeLa cells were tri-transfected with the indicated plasmids and, after 48 h, stained with anti-HA (red). GFP or GFP-TRIM44 expression is shown in green, while mCherry or mCherry-SQSTM1 is depicted in magenta. Scale bars measure 10 μm. (**B**) Quantification of cells with oligomerized SQSTM1: The percentage of cells displaying oligomerized SQSTM1 was determined through blind counting. The results, presented as the mean plus SD from three separate counts, indicate statistical significance at * *p* < 0.05 and *** *p* < 0.001 (unpaired *t*-tests). (**C**) Measurement of oligomerized SQSTM1 volume: The volume of oligomerized SQSTM1 was quantified, showing statistical significance with * *p* < 0.05 and *** *p* < 0.001 (unpaired *t*-tests). (**D**) Flow cytometry analysis: HeLa cells, including those with TRIM44 knockdown control (TRIM44[KD-CON]), TRIM44 knockdown (TRIM44[KD]), TRIM44 overexpression control (TRIM44[OE-CON]), and TRIM44 overexpression (TRIM44[OE]), were transfected as indicated with GFP or GFP-TRIM21. Following treatment with or without As[III] (5 µM, 24 h), apoptosis was assessed using Annexin V/7-AAD staining. These experiments were conducted three times, with data presented as means ± SD. Significance levels are denoted by * *p* < 0.05, ** *p* < 0.01, *** *p* < 0.001.

TRIM21 is essential in redox regulation, directly interacting with and ubiquitinating SQSTM1 at lysine 7 (K7) via K63-linkage through K63-linkage. This ubiquitination process hinders SQSTM1 oligomerization and its ability to sequester client proteins, including KEAP1, a negative regulator of the antioxidant response. Given this context, we investigated if TRIM44 could negate TRIM21’s suppression of the SQSTM1-Keap1-Nrf2 antioxidant pathway. We co-transfected cells with HA-SQSTM1, TRIM21, and TRIM44 or GFP as control and analyzed SQSTM1 oligomerization through immunofluorescence staining (Figure 7A). The percentage of cells with oligomerized SQSTM1 (Figure 7B) and volumes of oligomerized SQSTM1 (Figure 7C) significantly decreased in cells transfected with TRIM21 alone compared with control cells. However, these effects were nullified in cells co-transfected with TRIM44. Furthermore, As[III]-induced cell death was markedly reduced in cells co-transfected with TRIM21 and TRIM44 compared to those expressing TRIM21 only (Figure 7D left). Conversely, the knockdown of TRIM44 increased apoptosis induced by As[III] in the presence of TRIM21 expression (Figure 7D, right). Our findings support that TRIM44 effectively counteracts TRIM21’s negative regulation of the SQSTM1-Keap1-Nrf2 antioxidant pathway, thereby enhancing survival under oxidative stress.

## 4. Discussion

TRIM proteins play significant roles in a wide array of cellular processes, and there is growing evidence linking members of the TRIM family to the development and progression of various tumor types [42]. Research has shown that TRIM proteins are associated with several critical clinicopathological features and the prognosis of cancer patients [43]. Specifically, TRIM14, TRIM25 [44], TRIM32 [45], TRIM44, TRIM59 [46], and TRIM29 [47] exhibit abnormal expression in different cancer types and have been linked to patient prognosis. TRIM44, in particular, is upregulated in several cancers, including head and neck squamous cell carcinoma [48], lung [49], prostate [50], ovarian [51], and hepatocellular carcinoma [52]. Its roles vary from promoting cell migration and invasion to increasing drug resistance in cancer cells [52]. Additionally, elevated levels of TRIM44 are associated with poor prognosis in testicular germ cell tumors [53], esophageal squamous cell carcinoma [54], and gastric [55] and breast [56] cancers. In multiple myeloma (MM), TRIM44 is overexpressed in the osteoblastic niche of the bone marrow, allowing MM cells to compete with hematopoietic stem cells for niche support. While TRIM44 expression in MM cells increases bone destruction in xenograft mice, mirroring observations in MM patients [15], the mechanisms by which TRIM44 contributes to drug resistance in MM remain unclear.

In this report, we have identified TRIM44 as a novel regulator in the autophagy dynamics of MM. In addition, we discovered that TRIM44 is a potential prognostic marker for MM, with high levels of TRIM44 being associated with reduced overall survival among MM patients. This underscores its impact not only on the cellular mechanisms but also on patient outcomes. Our analyses of single-cell RNA-sequencing data from MM patients treated with Bortezomib-based therapies revealed that TRIM44 expression is markedly higher in suboptimal responders compared to those who respond optimally. Pathway analyses reveal that genes upregulated in these TRIM44-high, suboptimal responders are involved in the UPR, a critical factor in Bortezomib resistance. Our results highlight TRIM44’s new role in mediating resistance to Bortezomib treatment.

Autophagy is a cellular process that involves the delivery of abnormal, malfunctioning, or excessive cytoplasmic material to lysosomes for degradation. This process occurs at basal levels, playing a critical role in maintaining cellular homeostasis, and is implicated in numerous physiological and pathological processes [57]. Autophagy has been recognized as a multifunctional pathway activated in response to microenvironmental stress and intracellular damage caused by factors such as hypoxia, chemotherapeutic agents, viral infections, and toxins [58]. It can prevent the activation of apoptosis [59]. Furthermore, several multidrug-resistant cancers have been reported to exhibit upregulated autophagy biomarkers, highlighting the complex role of autophagy in cancer resistance mechanisms [60].

Recent reports have shown that several TRIM proteins regulate autophagy. A recent comprehensive analysis using HeLa cells has shown that several TRIM proteins function as autophagy receptors and regulators of autophagosome formation. Knockdown of 21 different TRIM proteins reduced LC3B punctum formation [61]. Several TRIM proteins interact with cargo/target-recognizing proteins, such as SQSTM1 and core regulators of autophagy, and form protein complexes called ‘TRIMosomes’ [42]. Here, we discovered a novel mechanism that TRIM44 promotes MM survival after therapy by enhancing autophagy. TRIM44 promotes SQSTM1 oligomerization and accelerates its degradation in a reactive oxygen species (ROS)-dependent manner under oxidative stress conditions in MM cells. Oligomerization is crucial for SQSTM1’s phosphorylation at S349, enabling TRIM44-mediated SQSTM1 oligomerization to sequester KEAP1 effectively, thereby boosting NFE2L2’s cytoprotective activities. This mechanism affords MM cells a robust defense against oxidative stress, underscoring the intricate balance between autophagy and survival pathways in MM cells.

The inhibition of the 26S proteasome by Bortezomib leads to the accumulation and aggregation of misfolded proteins in the ER lumen, which in turn activates the UPR via three key ER-resident transmembrane proteins: PERK, IRE1, and ATF6 [6]. PERK, a member of a family of protein kinases, phosphorylates the α subunit of the cytosolic eukaryotic translation initiation factor eIF2α. This phosphorylation results in reduced global protein synthesis and the preferential translation of selected mRNAs, including ATF4 [7]. ATF4 initiates a gene expression program that is involved in oxidative stress, amino acid synthesis, differentiation, metastasis, and angiogenesis, collectively known as integrated stress response [7]. Previous studies have identified ER stress and the eIF2α/PERK pathway as potent inducers of autophagy, promoting cell survival [62]. For example, Milani et al. reported that Bortezomib treatment increases UPR and autophagy activities [8]. Autophagy serves as a compensatory mechanism to eliminate Bortezomib-induced ROS and to counter ER stress-mediated apoptosis [38]. Our findings demonstrate that TRIM44 overexpression increases the expression levels of ATF4, phosphor-EIF2α, and LC3BII in cells treated with Bortezomib, indicating that TRIM44 expression enhances the effects of Bortezomib treatment on UPR and autophagy.

We also uncovered a new role for TRIM44 in counteracting the actions of TRIM21, the E3 ligase for SQSTM1, which negatively impacts SQSTM1-mediated sequestration of KEAP1 and the subsequent antioxidant response. Mechanistically, TRIM21 directly binds to and ubiquitylates SQSTM1 at the K7 residue. This modification prevents SQSTM1 dimerization and its sequestration function, which is crucial for its activity. The K7 residue is known to form a hydrogen bond with D69 [63], a bond vital for SQSTM1 dimerization [64]. In the absence of TRIM21, cells exhibit increased SQSTM1 oligomerization and aggregation, particularly under proteotoxic and oxidative stresses, similar to TRIM44 overexpressing cells.

This results in an enhanced antioxidant response and protection against superoxide-induced cell death and tissue damage. Given TRIM44’s protective role, we investigated the interaction between TRIM21 and TRIM44 under oxidative stress conditions. Introducing TRIM44 significantly reduced cell death triggered by arsenic trioxide (As[III]), effectively countering the suppressive effects of TRIM21. This interaction between TRIM proteins suggests potential therapeutic avenues, especially in scenarios marked by oxidative stress after Bortezomib treatments. Our study further explores the sensitivity of SQSTM1 to ROS-induced oxidation, underlining its significance in autophagy and survival pathways. The non-canonical activation of NRF2 via the SQSTM1-KEAP1-NRF2 axis, regulated by post-translational modifications of SQSTM1, highlights the intricate cellular responses to oxidative stress [65].

Oxidative stress represents a complex and dynamic condition marked by an imbalance between the production of ROS and the availability and action of antioxidants [66]. Bortezomib, through its proteasome inhibition mechanism, has been shown to induce oxidative stress in cancer cells [67]. Notably, cancer cells exhibit a greater susceptibility to damage from oxidative stress compared to normal cells due to their lower tolerance thresholds for redox homeostasis. This suggests that modulating ROS levels could be a viable strategy for achieving selective anticancer effects [68]. The generation of ROS in the context of Bortezomib treatment is believed to result from the accumulation of misfolded proteins that are typically degraded by the proteasome. The subsequent attempts at protein refolding led to elevated levels of ROS [69]. Studies have shown that ROS scavengers can mitigate, and the depletion of glutathione can enhance, Bortezomib-induced cell death in vitro, highlighting the significant role of ROS in the cytotoxic effects of Bortezomib [70]. The generation of ROS is now recognized as one of the early and crucial events triggering apoptotic signaling induced by Bortezomib in certain human cancer cell lines.

Our findings introduce a compelling challenge to the established understanding of mTORC1’s role in phosphorylating SQSTM1 at S349. Contrary to expectations, we noted an increase in SQSTM1 phosphorylation at S349 in TRIM44-overexpressing MM cells despite a marked decrease in mTOR activity. This anomaly led us to identify protein kinase A (PKA) as a previously unrecognized kinase responsible for this phosphorylation, thereby connecting the cAMP/PKA pathway to the KEAP1–NRF2 pathway and autophagy. This discovery is particularly significant in the context of Bortezomib treatment, where the observed resistance, associated with the upregulation in the UPR and autophagy, underscores TRIM44’s protective role against oxidative stress in MM cells. Our identification of PKA as the kinase targeting SQSTM1 S349 in response to oxidative stress unveils a novel mechanism through which TRIM44 modulates the SQSTM1-KEAP1-NRF2 axis.

In summary, our research highlights the complex roles of TRIM44 in MM, including its influence on autophagy dynamics, chemoresistance, and patient prognosis. Given its extensive impact, targeting TRIM44 emerges as a promising therapeutic strategy in MM, offering potential solutions to overcome resistance to existing treatments and providing new avenues for addressing MM.

## Data Availability

The original contributions presented in the study are included in the article/Supplementary Material, further inquiries can be directed to the corresponding author.

## Supplementary Materials

The following supporting information can be downloaded at: https://www.mdpi.com/article/10.3390/cells13171431/s1, Figure S1: TRIM44 is upregulated in t(4;14) positive multiple myeloma cell lines. Figure S2: Signatures of genes upregulated in TRIM44-high plasma cells from Optimal Responders. Figure S3: TRIM44 upregulation in Bortezomib-treated MM cells enhances the anti-MM activity of Bortezomib via UPR upregulation. Figure S4: Genes related to the resistance of MM cells to Bortezomib were upregulated in patients with TRIM44-high expression. Figure S5: Analysis of TRIM44 expression and apoptotic response in U266 cells treated with Carfilzomib.
