# Supplementary material for: TRIM44, a Novel Prognostic Marker, Supports the Survival of Proteasome-Resistant Multiple Myeloma Cells
Source: Cells. 2024 Aug 26;13(17):1431. doi: 10.3390/cells13171431 (PMC11394402; doi:10.3390/cells13171431)

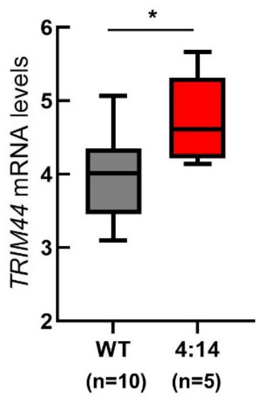

Figure S1. Boxplots showing normalized expression of TRIM44 in t(4;14) positive multiple myeloma (MM) cell lines (n=5) and t(4;14) negative MM cell lines (n=10).

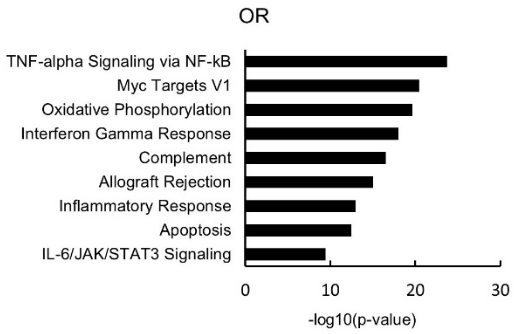

Figure S2. Bar plots illustrate the analysis of hallmark gene sets (from the Molecular Signatures Database, mSigDB) based on upregulated differentially expressed genes (DEGs) in TRIM44-high plasma cells from ORs.

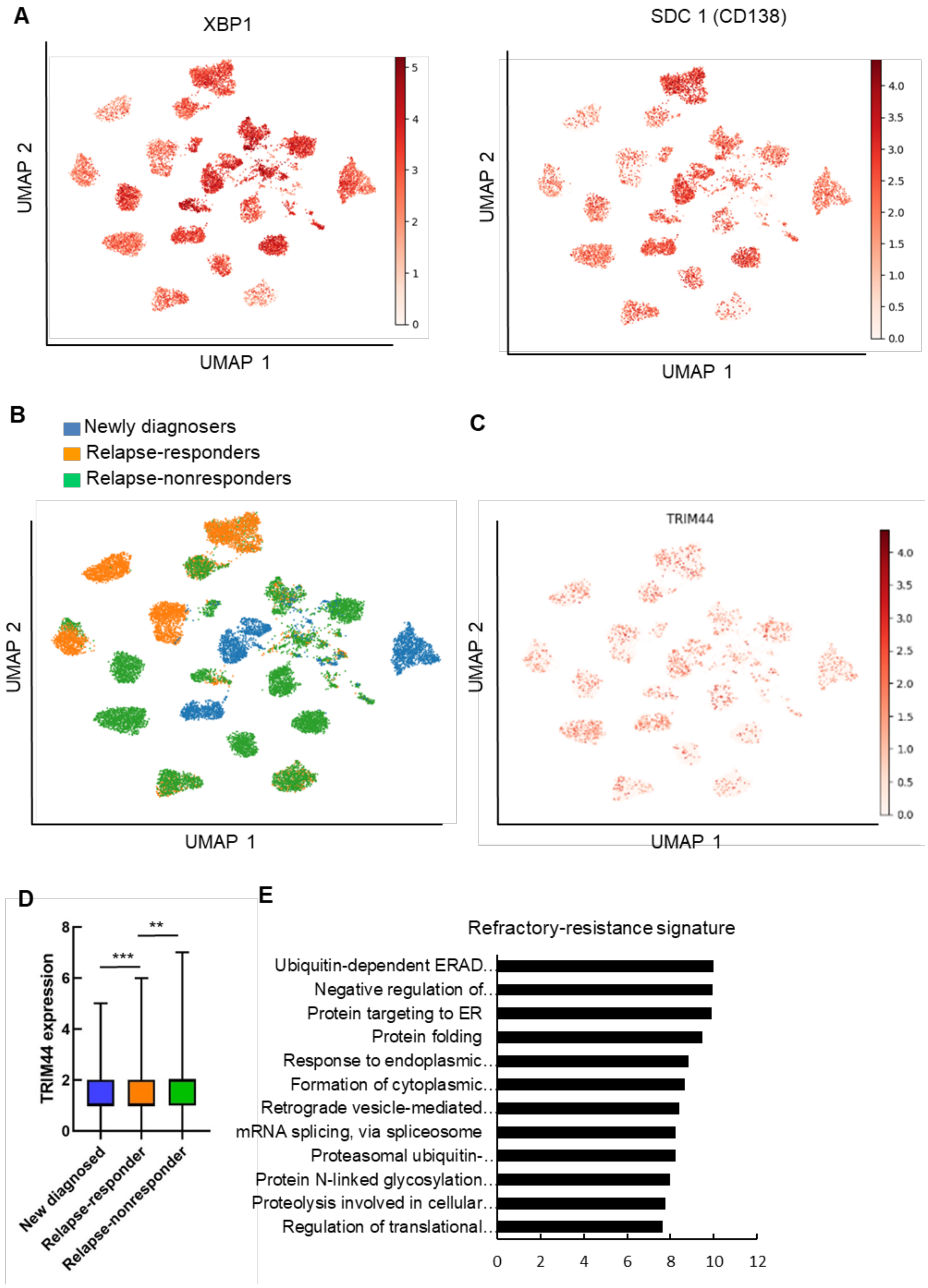

Figure S3. Single-cell RNA sequencing of CD138 positive plasma cells was analyzed, using data from the GSE189460 dataset. **A.** UMAP projection showing major clusters of cells from newly diagnosed patients, relapsed non-responders, and relapsed responders based on 2 markers of plasma cells, XBP1 and SDC1

(CD138). **B**. Respective cell-type assignments with the same embedding in A. **C**. UMAP projection showing RNA expression (log-normalized) of TRIM44. **D**. Boxplots showing normalized expression of TRIM44 in plasma cells of newly diagnosed patients, relapsed responders, and relapsed non-responders. **E**. Bar plots illustrate the analysis of hallmark gene sets (from the Molecular Signatures Database, mSigDB) based on upregulated differentially expressed genes (DEGs) in TRIM44-high plasma cells from relapsed non-responders.

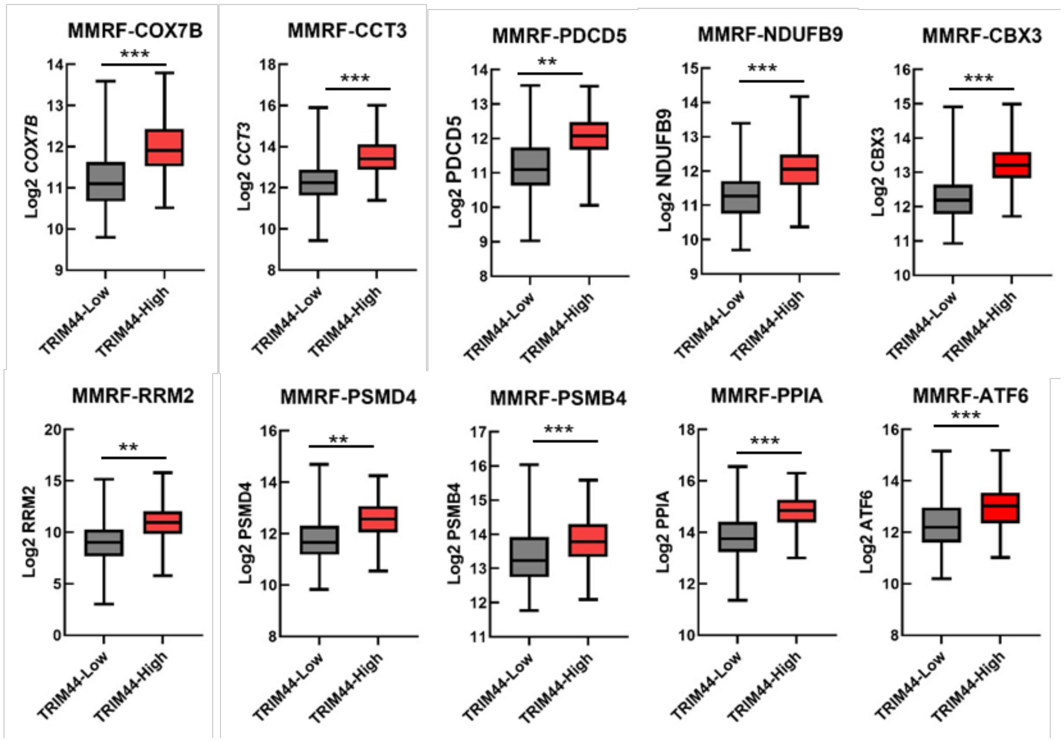

Figure S4. The MMRF RNA-sequencing data was downloaded from the Genomic Data Commons data portal. MM patients were divided into TRIM44-high and TRIM44-low expression groups. The top 25% of samples expressing TRIM44 were categorized as the high group, while the bottom 25% were classified as the low group. The mRNA expression levels of COX7B, CCT3, PDCD5, NDUFB9, CBX3, RPM2, PSMD4, PPIA, and ATF6 genes were compared between TRIM44-high and TRIM44-low expression groups. Significance levels are denoted as $**P < 0.01$, $***P < 0.001$ (unpaired t-tests).

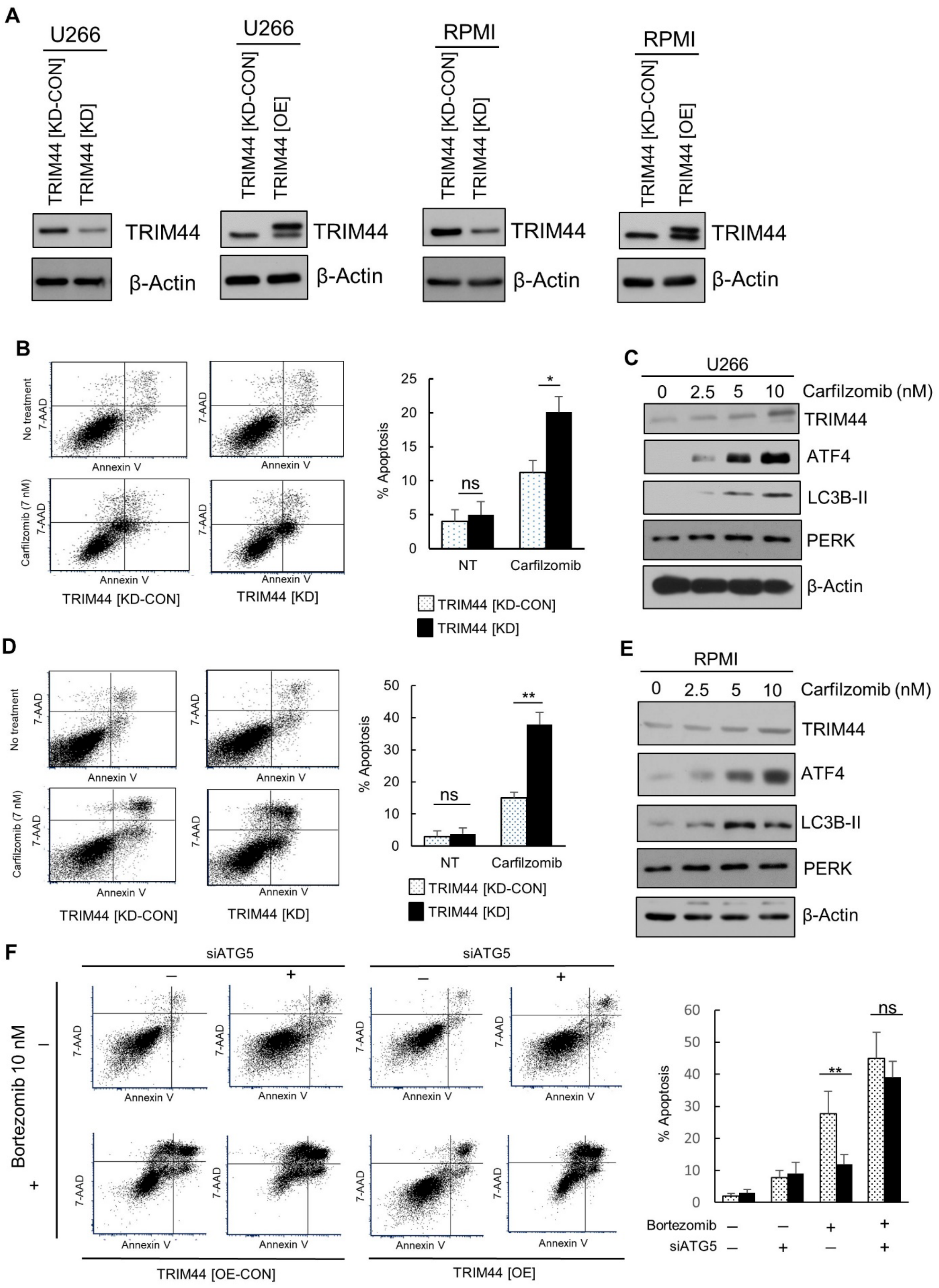
A
U266
TRIM44 [KD-CON]
TRIM44 [KD]
TRIM44
β-Actin
U266
TRIM44 [KD-CON]
TRIM44 [OE]
RPMI
TRIM44 [KD-CON]
TRIM44 [KD]
RPMI
TRIM44 [KD-CON]
TRIM44 [OE]
B
No treatment
Carfilzomib (7 nM)
7-AAD
Annexin V
TRIM44 [KD-CON]
TRIM44 [KD]
% Apoptosis
ns
*
NT
Carfilzomib
C
U266
0 2.5 5 10 Carfilzomib (nM)
TRIM44
ATF4
LC3B-II
PERK
β-Actin
D
**
E
RPMI
F
siATG5
Bortezomib 10 nM
TRIM44 [OE-CON]
TRIM44 [OE]
Bortezomib
siATG5

Figure S5. (A) Expression levels of TRIM44 in TRIM44 knockdown and control U266 and RPMI cells, as well as in TRIM44 overexpressing and control U266 and RPMI cells, were analyzed by Western blotting. **(B, D)** Flow Cytometry Analysis of Apoptosis: U266 cells were treated with 7 nM Carfilzomib for 24 hours and subsequently stained with PE/7-AAD. The percentage of apoptotic cells was quantified. Significance levels are indicated as **P < 0.01, ***P < 0.001 (unpaired t-tests). **(C, E)** Western Blot Analysis of TRIM44 Protein Levels: U266 (B) and RPMI (D) cells were exposed to Carfilzomib at the indicated concentrations for 24 hours. TRIM44 protein levels were then analyzed by Western blot using specific antibodies against TRIM44. **(F)** Flow Cytometry Analysis of Apoptosis with Autophagy Inhibition: U266 cells were transfected with siRNAs targeting ATG5 and treated with 10 nM Bortezomib for 24 hours, followed by staining with PE/7-AAD. The percentage of apoptotic cells was quantified. Significance levels are denoted as **P < 0.01, ***P < 0.001 (unpaired t-tests).

Original western blot:

Figure 4B

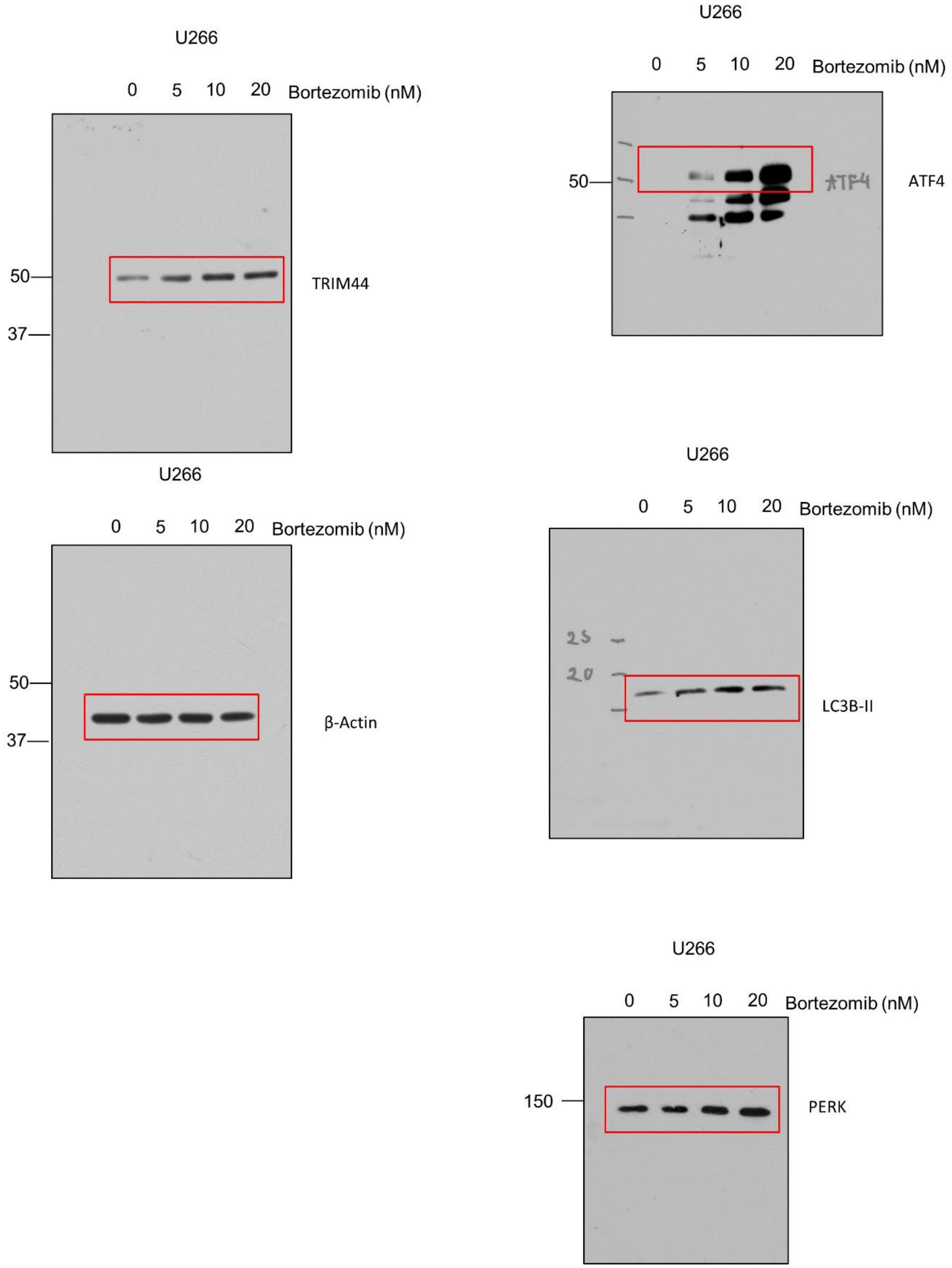

Figure 4D

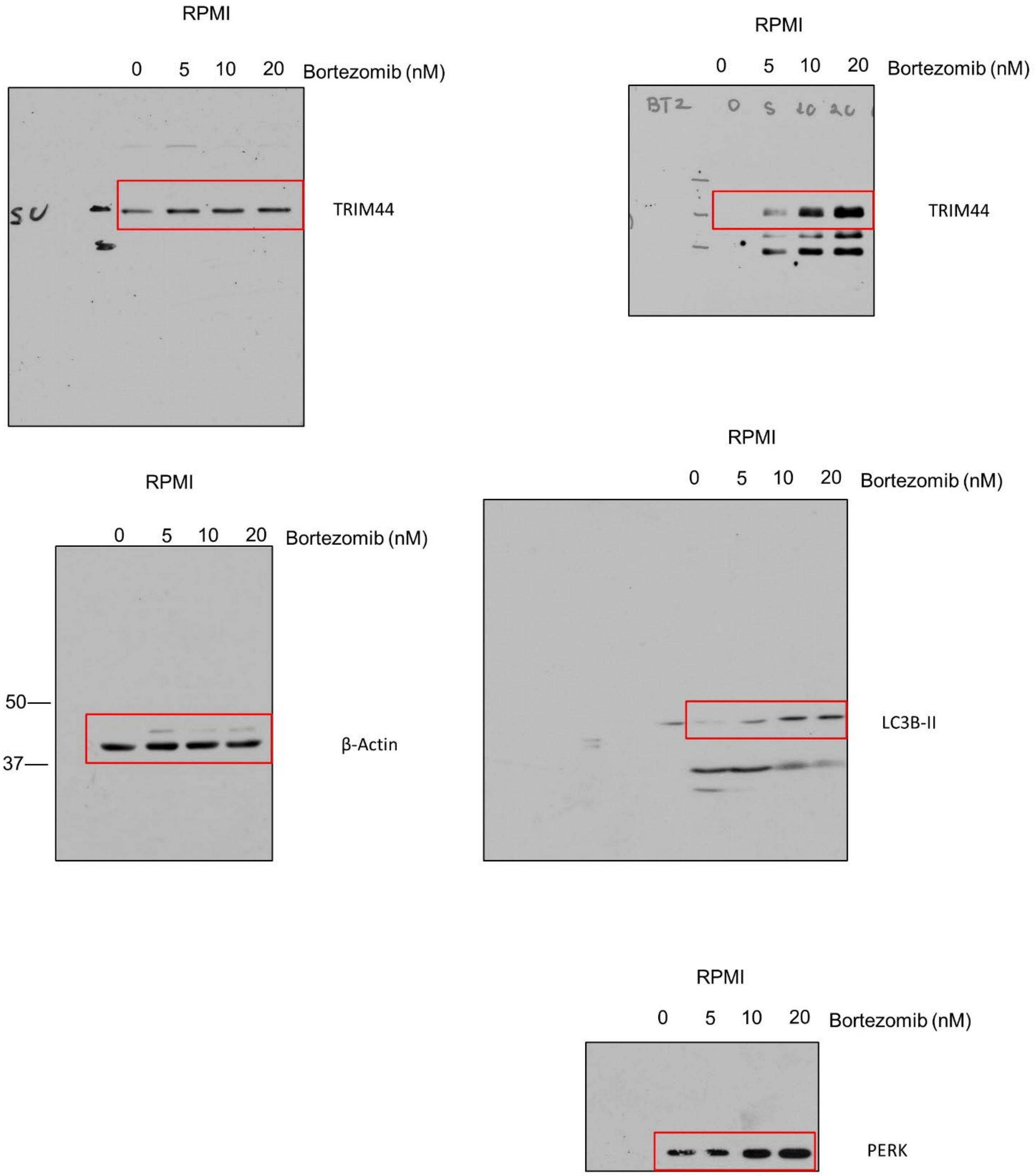
RPMI
0 5 10 20 Bortezomib (nM)
TRIM44
RPMI
0 5 10 20 Bortezomib (nM)
TRIM44
RPMI
0 5 10 20 Bortezomib (nM)
50—
37—
β-Actin
RPMI
0 5 10 20 Bortezomib (nM)
LC3B-II
RPMI
0 5 10 20 Bortezomib (nM)
PERK

Figure 4E - Left panels

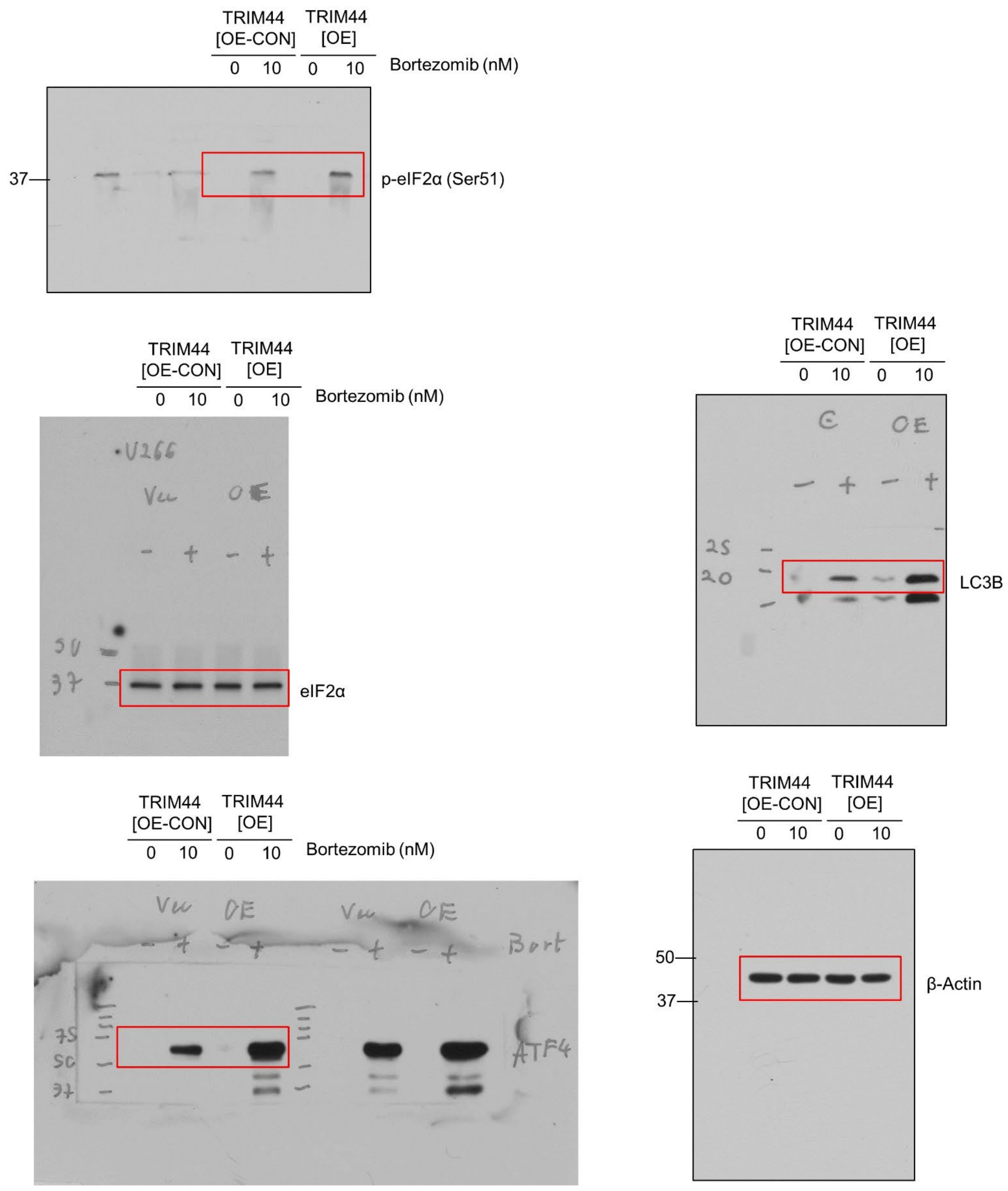

Figure 4C - Right panels

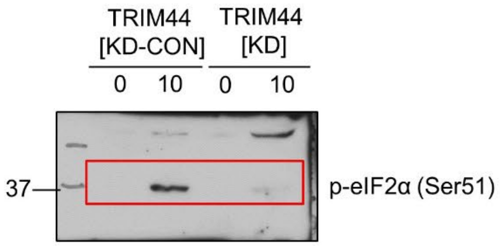

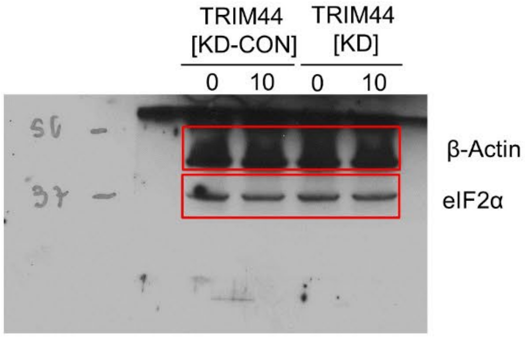

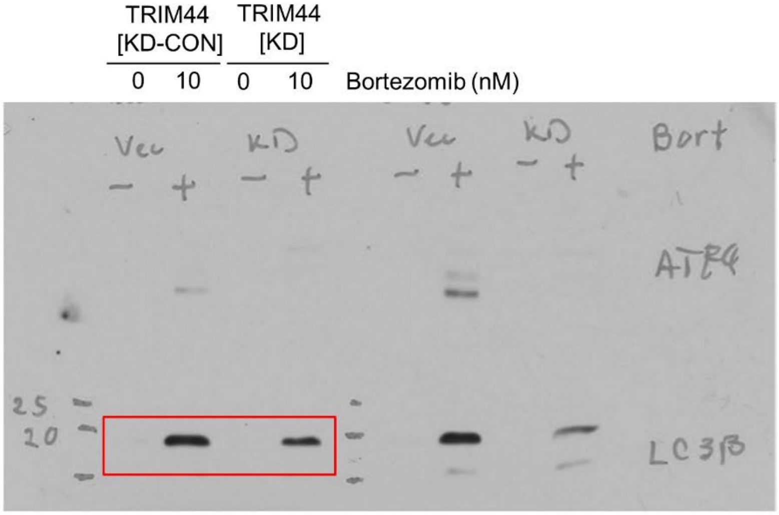

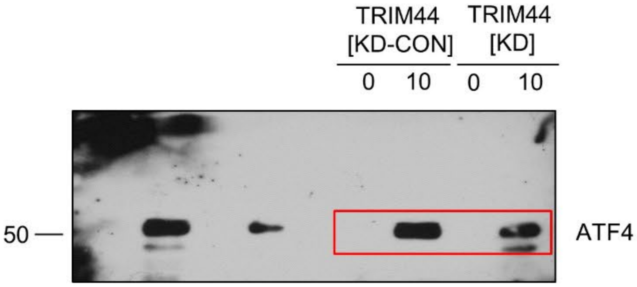

Figure 5 C

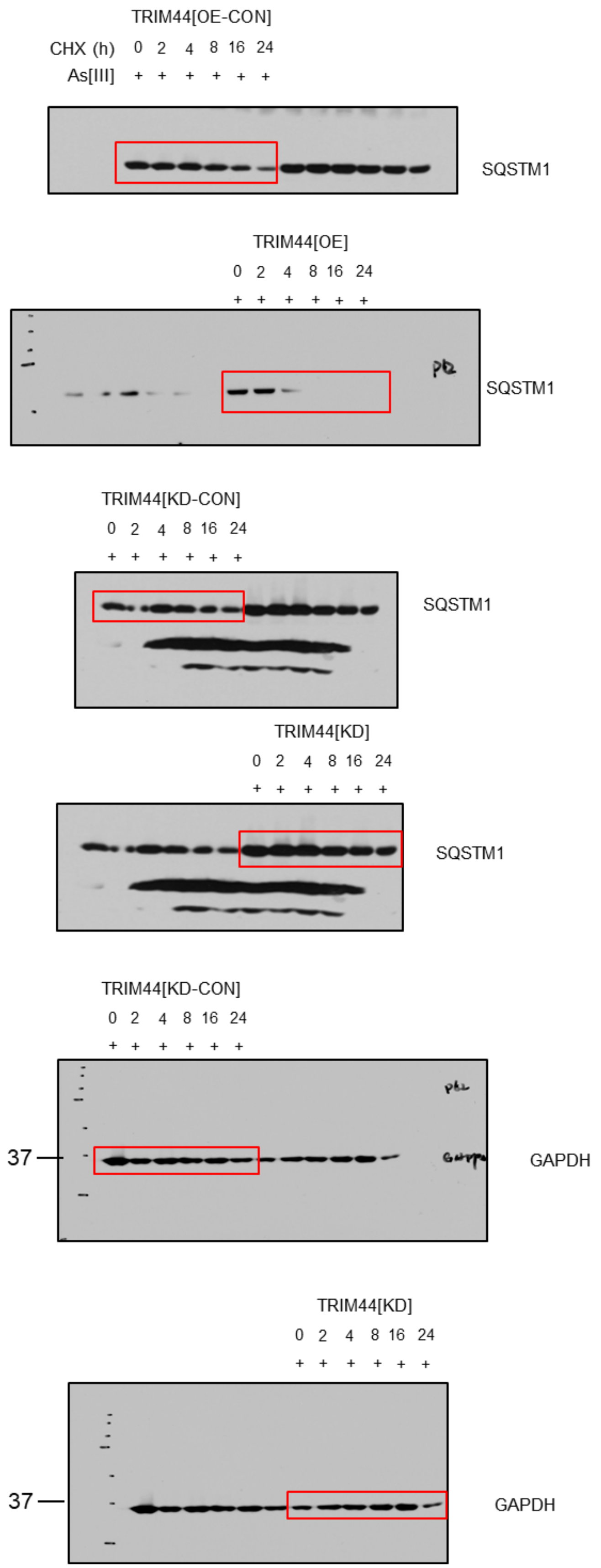
TRIM44[OE-CON]
CHX (h) 0 2 4 8 16 24
As[III] + + + + + +
SQSTM1
TRIM44[OE]
0 2 4 8 16 24
+ + + + + +
SQSTM1
TRIM44[KD-CON]
0 2 4 8 16 24
+ + + + + +
SQSTM1
TRIM44[KD]
0 2 4 8 16 24
+ + + + + +
SQSTM1
TRIM44[KD-CON]
0 2 4 8 16 24
+ + + + + +
37
GAPDH
TRIM44[KD]
0 2 4 8 16 24
+ + + + + +
37
GAPDH

Figure 5 E

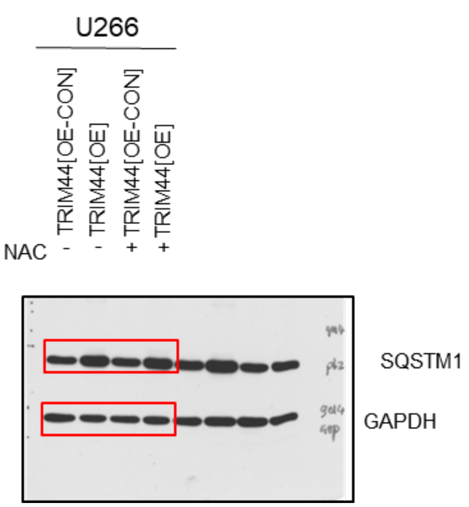
U266
TRIM44[OE-CON]
TRIM44[OE]
TRIM44[OE-CON]
TRIM44[OE]
NAC - - + +
SQSTM1
GAPDH

Figure 5G

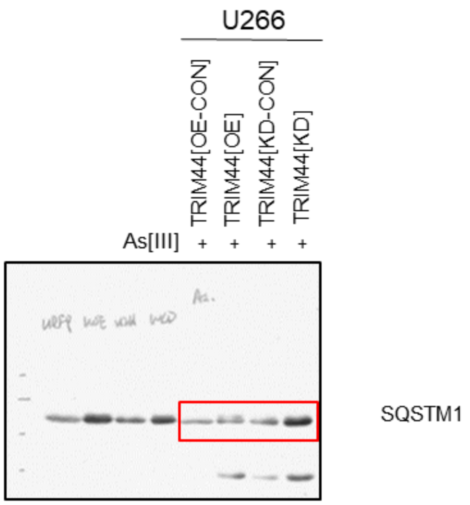
U266
TRIM44[OE-CON]
TRIM44[OE]
TRIM44[KD-CON]
TRIM44[KD]
As[III] + + + +
SQSTM1

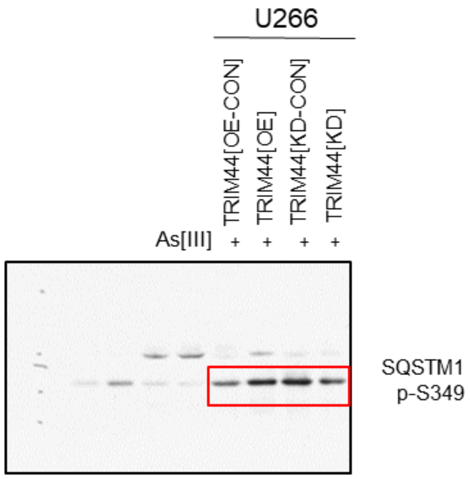
U266
TRIM44[OE-CON]
TRIM44[OE]
TRIM44[KD-CON]
TRIM44[KD]
As[III] + + + +
SQSTM1
p-S349

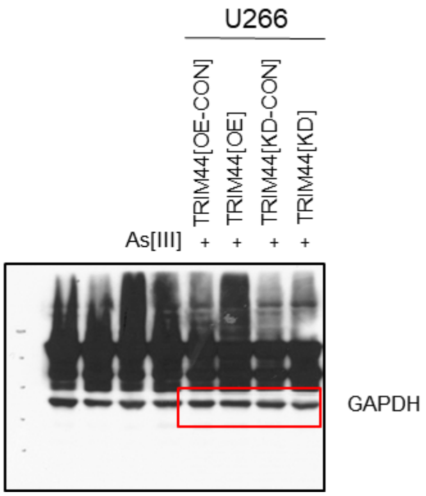
U266
TRIM44[OE-CON]
TRIM44[OE]
TRIM44[KD-CON]
TRIM44[KD]
As[III] + + + +
GAPDH

Figure 5H

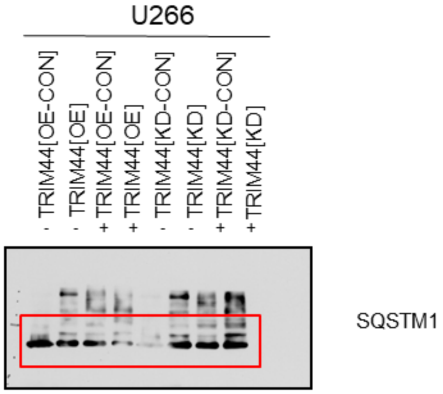
U266
- TRIM44[OE-CON]
- TRIM44[OE]
+ TRIM44[OE-CON]
+ TRIM44[OE]
- TRIM44[KD-CON]
- TRIM44[KD]
+ TRIM44[KD-CON]
+ TRIM44[KD]
SQSTM1

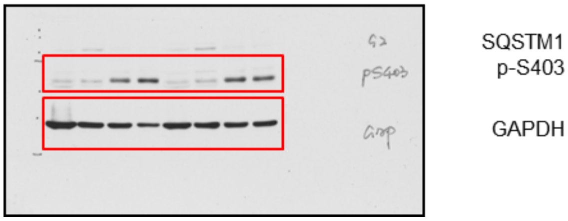
SQSTM1
p-S403
GAPDH

Figure 6 A

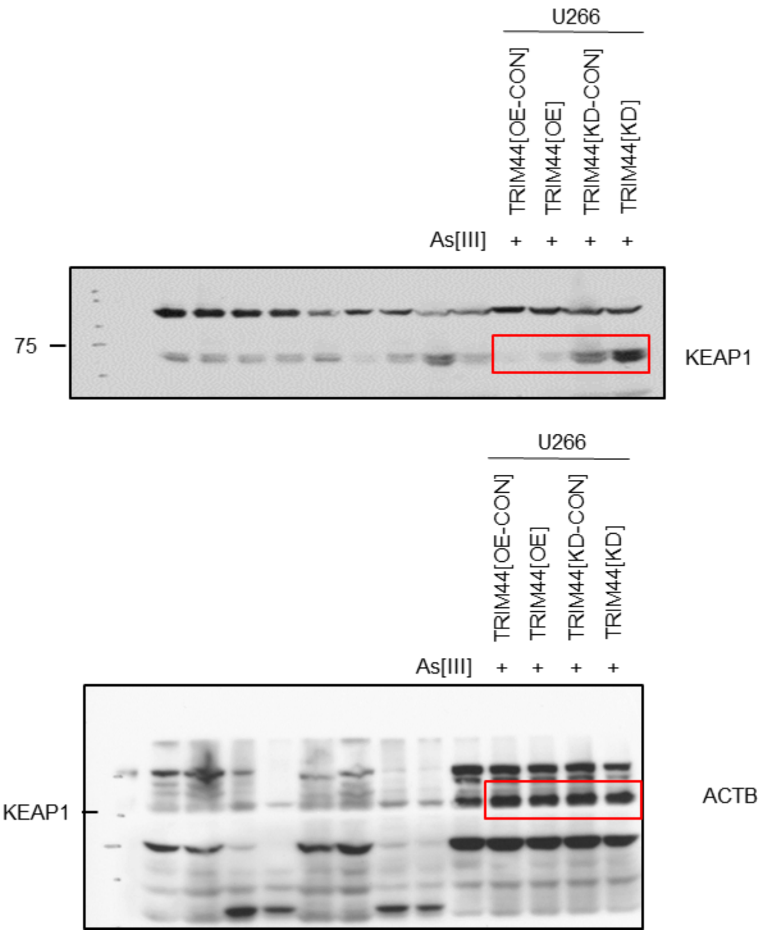
U266
TRIM44[OE-CON]
TRIM44[OE]
TRIM44[KD-CON]
TRIM44[KD]
As[III]
+
+
+
+
75
KEAP1
U266
TRIM44[OE-CON]
TRIM44[OE]
TRIM44[KD-CON]
TRIM44[KD]
As[III]
+
+
+
+
KEAP1
ACTB

Figure 6B

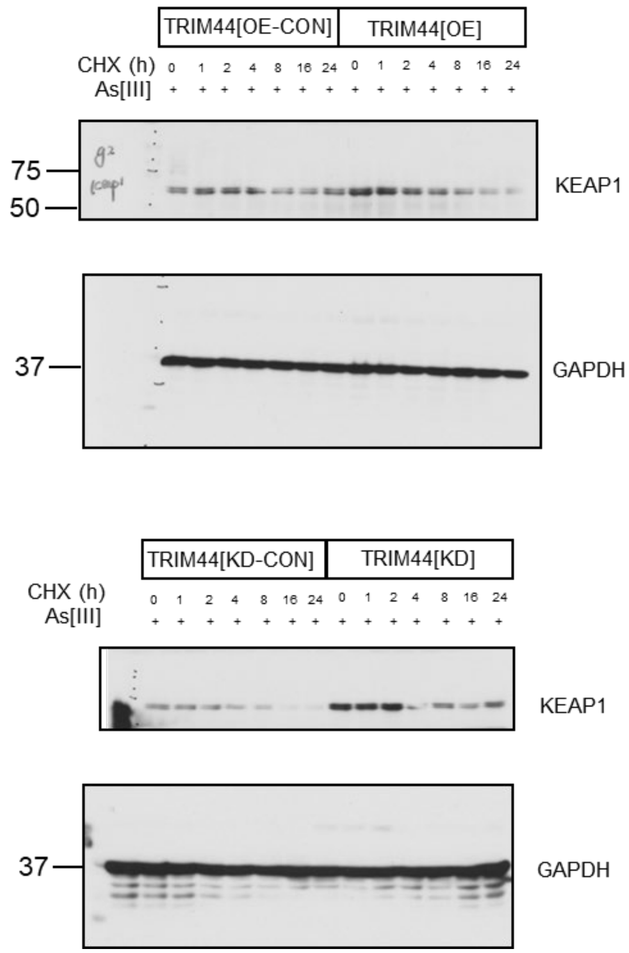
TRIM44[OE-CON]
TRIM44[OE]
CHX (h)
0 1 2 4 8 16 24 0 1 2 4 8 16 24
As[III]
75
50
KEAP1
37
GAPDH
TRIM44[KD-CON]
TRIM44[KD]
CHX (h)
0 1 2 4 8 16 24 0 1 2 4 8 16 24
As[III]
KEAP1
37
GAPDH

Figure 6E

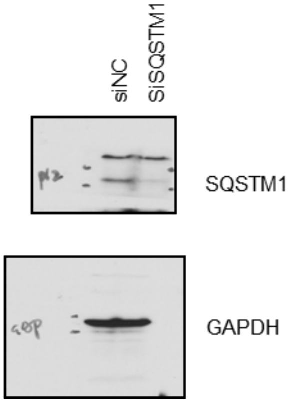

Supplement: Supplementary file 1 [file cells-13-01431-s001.zip › cells-3112242-supplementary.pdf]
